# Supplementary material for: Shifting research priorities in maternal and child health in the COVID-19 pandemic era in India: A renewed focus on systems strengthening
Source: PLoS One. 2021 Aug 12;16(8):e0256099. doi: 10.1371/journal.pone.0256099 (PMC8360530; doi:10.1371/journal.pone.0256099)
Supplement: S1 File — (DOCX) [file pone.0256099.s001.docx]

**S1 File. Research Needs in Maternal and Child Health Survey: India**

**Start of Block: Survey Questions**

 
**Introduction**

As part of a collaborative initiative lead by the Johns Hopkins Bloomberg School of Public Health, a Maternal and Child Health Center India (MCHI) is being established in Kolkata, West Bengal starting this year.

We are planning to focus our resources on assisting India in reaching the sustainable development goals (SDGs) related to MCH (Maternal and Child Health), with particular focus on:

- reducing infant and child mortality and improving child quality of life
- improving maternal health
- combating common and emerging infectious diseases

As part of our planning and evaluation of the public health landscape in maternal and child health in India, we are conducting this survey to identify key research priorities.

Responding to this survey request is voluntary; it is your choice.  Completing this survey and submitting it would mean that you consent to participate in the study.

You may choose not to answer any question that we ask.

Please reflect on your current understanding of India’s capacity to address these issues as you answer the questions below.

Q1 **Please tell us about yourself**

Location (specify city and state)

________________________________________________________________

Q2 **Profession**

- Public health research
- Lab research
- Healthcare worker (Doctor, Nurse, CHW, Social Worker, Psychologist, Other): _____
- Policy- maker
- Teaching
- Other, specify: ________________________________________________

Q3 **Primary Area of Work**

- Health policy
- Family planning
- Child health
- Maternal health
- Engineering/Infrastructure
- Infectious Disease
- Biostats/Epidemiology
- Other, Specify: ________________________________________________

Q4 **Number of years worked in primary area of work:**

________________________________________________________________

Q5 **Institution or Organization**:

________________________________________________________________

Q6 **Type of Organization (Primary)**

- Government
- Non-government
- Private organization
- University
- Other, specify: ________________________________________________

Q7   **Vaccine Preventable Diseases**

In considering vaccine preventable diseases, please rate the importance of the following topics as a research priority for maternal and child health in India.

|  | Extremely important | Very important | Moderately important | Slightly important | Not at all important |
| --- | --- | --- | --- | --- | --- |
| Strengthening the public sector workforce |  |  |  |  |  |
| Areas of disparity, such as vaccination rates among the urban poor |  |  |  |  |  |
| Assisting state government initiatives for vaccine delivery |  |  |  |  |  |
| Strengthening advocacy efforts around newer vaccines/vaccine introduction |  |  |  |  |  |
| Improving vaccination coverage and research opportunities |  |  |  |  |  |
| Communications on vaccine use and addressing vaccine hesitancy |  |  |  |  |  |
| Research focused on reaching 'Zero Dose' children |  |  |  |  |  |
| Role of vaccines in preventing other healthcare outcomes (eg: Antimicrobial resistance) |  |  |  |  |  |
| Enhancing public private partnerships for vaccine delivery |  |  |  |  |  |
| Other, specify: |  |  |  |  |  |

Q8 **Ranking Vaccine Preventable Disease Research Topics**
 Please rate the topics as described above in priority order, with 8=lowest priority and 1=highest priority.  (Click each option and then drag them into your desired ranking order)

______ Strengthening the public sector workforce

______ Areas of disparity, such as vaccination rates among the urban poor

______ Assisting state government initiatives for vaccine delivery

______ Strengthening advocacy efforts around newer vaccines/vaccine introduction

______ Improving vaccination coverage to reach under immunized populations

______ Communications on vaccine use and addressing vaccine hesitancy

______ Enhancing public-private partnerships for vaccination delivery

______ Role of vaccines in preventing other healthcare outcomes (e.g.: antimicrobial resistance)

______ Other, specify:

Q9 **What other priorities not covered above should be considered for vaccine-preventable diseases?**

________________________________________________________________

________________________________________________________________

________________________________________________________________

________________________________________________________________

________________________________________________________________

Q10 **Outbreak Preparedness**
The public health workforce in India, and broad development goals, are likely to be impacted by the current COVID-19 pandemic.  Please rate the following topics as a research priority for

maternal and child health in India.

|  | Extremely important | Very important | Moderately important | Slightly important | Not at all important |
| --- | --- | --- | --- | --- | --- |
| Developing lab capacity and training |  |  |  |  |  |
| Enhancing public health surveillance networks |  |  |  |  |  |
| Conducting COVID-19 research for Maternal and Child Health sub-populations |  |  |  |  |  |
| Assisting state government initiatives for disease surveillance |  |  |  |  |  |
| Strengthening community knowledge and awareness of disease transmission |  |  |  |  |  |
| Enhancing infrastructure needs for disease management (eg: Oxygen, Modeling for healthcare service needs) |  |  |  |  |  |
| Other, specify: |  |  |  |  |  |

Q11   **Ranking Outbreak Preparedness Research Topics**
 Please rate the Outbreak Preparedness topics as described above in priority order, with 6=lowest priority and 1=highest priority.  (Click each option and then drag them into your desired ranking order)

______ Developing lab capacity and training

______ Enhancing public health surveillance networks

______ Conducting COVID-19 research for Maternal and Child Health sub-populations

______ Assisting state government initiatives for disease surveillance

______ Strengthening community knowledge and awareness of disease transmission

______ Enhancing infrastructure needs for disease management (eg: Oxygen, Modeling for healthcare service needs)

______ Other, specify:

Q12 **What other priorities not covered above should be considered for outbreak preparedness research?**

________________________________________________________________

________________________________________________________________

________________________________________________________________

________________________________________________________________

________________________________________________________________

Q13 **Primary Health Care Integration**

Considering the critical importance of Asha workers and anganwadi centers to primary health care delivery and integration, please rate the importance of the following topics as a research priority for maternal and child health in India.

|  | Extremely important | Very important | Moderately important | Slightly important | Not at all important |
| --- | --- | --- | --- | --- | --- |
| Nutrition education support through community workers |  |  |  |  |  |
| Supplemental nutrition delivery and effectiveness |  |  |  |  |  |
| Growth monitoring through regular check ups |  |  |  |  |  |
| Early childhood care or preschool |  |  |  |  |  |
| Supporting research around community service delivery and effectiveness |  |  |  |  |  |
| Other, Specify: |  |  |  |  |  |

Q14 **Ranking Primary Health Care Integration Research Topics**

Please rate the Primary Health Care Integration research topics as described above in priority order, with 6=lowest priority and 1=highest priority.  (Click each option and then drag them into your desired ranking order)

______ Nutrition education support through community workers

______ Supplemental nutrition delivery and effectiveness

______ Growth monitoring through regular check ups

______ Early childhood care or preschool

______ Supporting research around community service delivery and effectiveness

______ Other, specify:

Q15 **What other priorities not covered above should be considered for primary health care research?**

________________________________________________________________

________________________________________________________________

________________________________________________________________

________________________________________________________________

________________________________________________________________

Q16 **Maternal Health**
Considering progress in maternal health across India, please rate the importance of the following topics as a research priority for maternal and child health in India.

|  | Extremely important | Very important | Moderately important | Slightly important | Not at all important |
| --- | --- | --- | --- | --- | --- |
| Improving maternal immunization coverage |  |  |  |  |  |
| Encouraging at least 4-8 antenatal visits during pregnancy |  |  |  |  |  |
| Improving facility based safe delivery outcomes |  |  |  |  |  |
| Financial assistance/expenditures for births at an institution |  |  |  |  |  |
| Incentivizing use of Mother and Child Protection Cards |  |  |  |  |  |
| Improving maternal nutrition/supplementation (iron, folic acid supplementation) |  |  |  |  |  |
| Delivering family planning interventions at the time of childbirth |  |  |  |  |  |
| Other, Specify: |  |  |  |  |  |

Q17 **Ranking Maternal Health Research Topics** Please rate the Maternal Health research topics as described above in priority order, with 8=lowest priority and 1=highest priority.  (Click each option and then drag them into your desired ranking order)

______ Improving maternal immunization coverage

______ Encouraging at least 4-8 antenatal visits during pregnancy

______ Improving in-hospital delivery outcomes

______ Financial assistance/expenditures for births at an institution

______ Incentivizing use of Mother and Child Protection Cards

______ Improving maternal nutrition/supplementation (iron, folic acid supplementation)

______ Delivering family planning interventions at the time of childbirth

______ Other, specify:

Q18 **What other priorities not covered above should be considered for maternal health research?**

________________________________________________________________

________________________________________________________________

________________________________________________________________

________________________________________________________________

________________________________________________________________

Q19 **Neonatal Health**
Considering progress in neonatal health across India, please rate the importance of the following topics as a research priority for maternal and child health in India.

|  | Extremely important | Very important | Moderately important | Slightly important | Not at all important |
| --- | --- | --- | --- | --- | --- |
| Neonatal resuscitation to reduce perinatal asphyxia |  |  |  |  |  |
| Improving initiation of breast feeding in hospital |  |  |  |  |  |
| Prevention and management of newborn sepsis |  |  |  |  |  |
| Kangaroo mother care for low birth weight babies |  |  |  |  |  |
| Improving quality of care during labor and birth |  |  |  |  |  |
| Community-based extra care for preterm/low birthweight babies |  |  |  |  |  |
| Vaccinating the neonate prior to discharge from the facility |  |  |  |  |  |
| Enhancing early home based newborn care |  |  |  |  |  |
| Other, Specify: |  |  |  |  |  |

Q20 **Ranking Neonatal Health Research Topics**
 Please rate the Neonatal Health Integration research topics as described above in priority order, with 8=lowest priority and 1=highest priority.  (Click each option and then drag them into your desired ranking order)

______ Neonatal resuscitation to reduce perinatal asphyxia

______ Improving initiation of breast feeding in hospital

______ Prevention and management of newborn sepsis

______ Kangaroo mother care for low birth weight babies

______ Improving quality of care during labor and birth

______ Community-based extra care for preterm/low birthweight babies

______ Vaccinating the neonate prior to discharge from the facility

______ Other, specify:

Q21 **What other priorities not covered above should be considered for neonatal health research?**

________________________________________________________________

________________________________________________________________

________________________________________________________________

________________________________________________________________

________________________________________________________________

Q22 **Infectious Diseases**
How would you rank what would be the best use of MCHI resources for addressing India's infectious disease priorities?

|  | Extremely important | Very important | Moderately important | Slightly important | Not at all important |
| --- | --- | --- | --- | --- | --- |
| Dengue prevention and management |  |  |  |  |  |
| COVID-19 |  |  |  |  |  |
| Pediatric and maternal screening and treatment for tuberculosis |  |  |  |  |  |
| Maternal and neonatal screening for HIV |  |  |  |  |  |
| Pediatric and maternal screening and treatment for malaria |  |  |  |  |  |
| Pediatric and maternal screening for influenza and other viral illnesses |  |  |  |  |  |
| Diarrhoeal diseases prevention and treatment |  |  |  |  |  |
| Neglected tropical diseases (Hookworm, Dengue, Rabies, Ascariasis) |  |  |  |  |  |
| Other, specify: |  |  |  |  |  |

Q23 **Ranking Infectious Diseases Health Research Topics**
 Please rate the Infectious Diseases research topics as described above in priority order, with 8=lowest priority and 1=highest priority.  (Click each option and then drag them into your desired ranking order)

______ Dengue prevention and management

______ COVID-19

______ Pediatric and maternal screening and treatment for tuberculosis

______ Maternal and neonatal screening for HIV

______ Pediatric and maternal screening and treatment for malaria

______ Pediatric and maternal screening for influenza and other viral illnesses

______ Diarrhoeal diseases prevention and treatment

______ Neglected tropical diseases (Hookworm, Dengue, Rabies, Ascariasis)

______ Other, specify:

Q24 **What other priorities not covered above should be considered for infectious diseases research?**

________________________________________________________________

________________________________________________________________

________________________________________________________________

________________________________________________________________

________________________________________________________________

Q25 **Is there anything else you would advise as MCHI develops MCH-focused research priorities?** 
   ___________________________________________________________

________________________________________________________________

________________________________________________________________

________________________________________________________________

________________________________________________________________

Q26 **How do you think MCHI should communicate the findings of this baseline evaluation with stakeholders? Do you have suggestions for a specific format?**

- Journals
- Conferences
- Workshops
- Newsletter
- Other, Specify:

______________________________________________________________________

Thank you very much for taking the time to complete this survey.  Please leave your name and email address below so that we can contact you to do a mid-point and final evaluation of MCHI's programs.


Name:

________________________________________________________________

Email:

________________________________________________________________

We appreciate you taking the time to complete this survey. Please review your answers and click the following arrow to submit.
