# Supplementary material for: Shifting research priorities in maternal and child health in the COVID-19 pandemic era in India: A renewed focus on systems strengthening
Source: PLoS One. 2021 Aug 12;16(8):e0256099. doi: 10.1371/journal.pone.0256099 (PMC8360530; doi:10.1371/journal.pone.0256099)
Supplement: S2 File — (DOCX) [file pone.0256099.s002.docx]

**S2 File. Raw Data: Research Needs Assessment**

**Table of Contents**

1. **Likert**
   1. Frequencies
2. **Ranking (Reassigned)**
   1. Frequencies
   2. Mean weighed score
3. **“Other” write-in**
   1. Summary of Likert/Ranking Main “Other” Responses
   2. All Likert Responses
   3. All Ranking Responses
   4. Overall “Other”
4. **Demographics**
5. **Likert Frequencies**

**Vaccine Preventable Diseases**

| **Sub-domain** | **Extremely Important** | **Very Important** | **Moderately Important** | **Slightly Important** | **Not at all Important** |
| --- | --- | --- | --- | --- | --- |
| Strengthening the public sector workforce (n = 83) | 46 | 21 | 13 | 2 | 1 |
| Areas of disparity, such as vaccination rates among the urban poor (n = 84) | 47 | 26 | 8 | 3 | 0 |
| Assisting state government initiatives for vaccine delivery (n = 82) | 28 | 29 | 19 | 6 | 0 |
| Strengthening advocacy efforts around newer vaccines/vaccine introduction (n = 84) | 29 | 23 | 22 | 8 | 2 |
| Improving vaccination coverage and research opportunities (n = 82) | 45 | 28 | 8 | 1 | 0 |
| Communications on vaccine use and addressing vaccine hesitancy (n = 83) | 37 | 33 | 7 | 6 | 0 |
| Research focused on reaching 'Zero Dose' children (n = 82) | 23 | 41 | 12 | 6 | 0 |
| Role of vaccines in preventing other healthcare outcomes (eg: Antimicrobial resistance) ( n = 83) | 30 | 24 | 21 | 5 | 3 |
| Enhancing public private partnerships for vaccine delivery (n = 83) | 28 | 33 | 14 | 5 | 3 |

**Outbreak Preparedness**

| **Sub-Domain** | **Extremely Important** | **Very Important** | **Moderately Important** | **Slightly Important** | **Not at all Important** |
| --- | --- | --- | --- | --- | --- |
| Developing lab capacity and training (n = 84) | 42 | 33 | 8 | 1 | 0 |
| Enhancing public health surveillance networks (n = 82) | 63 | 15 | 4 | 0 | 0 |
| Conducting COVID-19 research for Maternal and Child Health sub-populations (n = 83) | 40 | 27 | 13 | 2 | 1 |
| Assisting state government initiatives for disease surveillance (n = 84) | 47 | 32 | 4 | 0 | 1 |
| Strengthening community knowledge and awareness of disease transmission (n = 84) | 44 | 30 | 8 | 2 | 0 |
| Enhancing infrastructure needs for disease management (eg: Oxygen, Modeling for healthcare service needs) (n = 84) | 49 | 23 | 10 | 2 | 0 |

**Primary Care Integration**

| **Sub-Domain** | **Extremely Important** | **Very Important** | **Moderately Important** | **Slightly Important** | **Not at all Important** |
| --- | --- | --- | --- | --- | --- |
| Nutrition education support through community workers (n = 82) | 52 | 20 | 8 | 1 | 1 |
| Supplemental nutrition delivery and effectiveness (n = 81) | 39 | 31 | 9 | 2 | 0 |
| Growth monitoring through regular check ups (n = 81) | 49 | 22 | 8 | 2 | 0 |
| Early childhood care or preschool (n = 82) | 43 | 33 | 6 | 0 | 0 |
| Supporting research around community service delivery and effectiveness (n = 82) | 50 | 23 | 7 | 1 | 1 |

**Maternal Health**

| **Sub-Domain** | **Extremely Important** | **Very Important** | **Moderately Important** | **Slightly Important** | **Not at all Important** |
| --- | --- | --- | --- | --- | --- |
| Improving maternal immunization coverage (n = 82) | 33 | 22 | 19 | 6 | 2 |
| Encouraging at least 4-8 antenatal visits during pregnancy (n = 83) | 45 | 22 | 15 | 1 | 0 |
| Improving facility based safe delivery outcomes (n = 83) | 53 | 23 | 5 | 2 | 0 |
| Financial assistance/expenditures for births at an institution (n = 83) | 28 | 25 | 24 | 4 | 2 |
| Incentivizing use of Mother and Child Protection Cards (n = 83) | 26 | 25 | 21 | 9 | 2 |
| Improving maternal nutrition/supplementation (iron, folic acid supplementation) (n = 83) | 50 | 26 | 5 | 2 | 0 |
| Delivering family planning interventions at the time of childbirth (n = 82) | 39 | 31 | 9 | 1 | 2 |

**Neonatal Health**

| **Sub-Domain** | **Extremely Important** | **Very Important** | **Moderately Important** | **Slightly Important** | **Not at all Important** |
| --- | --- | --- | --- | --- | --- |
| Neonatal resuscitation to reduce perinatal asphyxia (n = 84) | 57 | 17 | 9 | 1 | 0 |
| Improving initiation of breast feeding in hospital (n = 84) | 56 | 19 | 8 | 0 | 1 |
| Prevention and management of newborn sepsis (n = 83) | 51 | 23 | 7 | 2 | 0 |
| Kangaroo mother care for low birth weight babies (n = 84) | 53 | 24 | 6 | 1 | 0 |
| Improving quality of care during labor and birth  (n = 83) | 62 | 19 | 2 | 0 | 0 |
| Community-based extra care for preterm/low birthweight babies  (n = 83) | 56 | 22 | 5 | 0 | 0 |
| Vaccinating the neonate prior to discharge from the facility (n = 83) | 40 | 28 | 11 | 3 | 1 |
| Enhancing early home based newborn care  (n = 84) | 49 | 29 | 6 | 0 | 0 |

**Infectious Diseases**

| **Sub-Domain** | **Extremely Important** | **Very Important** | **Moderately Important** | **Slightly Important** | **Not at all Important** |
| --- | --- | --- | --- | --- | --- |
| Dengue prevention and management (n = 82) | 26 | 30 | 20 | 5 | 1 |
| COVID-19 (n = 83) | 38 | 24 | 16 | 5 | 0 |
| Pediatric and maternal screening and treatment for tuberculosis (n = 84) | 33 | 33 | 14 | 4 | 0 |
| Maternal and neonatal screening for HIV (n = 84) | 29 | 30 | 20 | 4 | 1 |
| Pediatric and maternal screening and treatment for malaria (n = 83) | 22 | 36 | 16 | 8 | 1 |
| Pediatric and maternal screening for influenza and other viral illnesses (n = 83) | 16 | 34 | 24 | 7 | 2 |
| Diarrhoeal diseases prevention and treatment (n = 84) | 53 | 21 | 7 | 3 | 0 |
| Neglected tropical diseases (Hookworm, Dengue, Rabies, Ascariasis)  (n = 83) | 17 | 35 | 19 | 11 | 1 |

1. **Ranking (Freq. and Mean Weighted Score)**

**Vaccine Preventable Diseases**

| **n = 80** | **Rankings** |  |  |  |  |  |  |  |  |  |
| --- | --- | --- | --- | --- | --- | --- | --- | --- | --- | --- |
| **Sub-domain** | **1** | **2** | **3** | **4** | **5** | **6** | **7** | **8** | **9** | **10** |
| Strengthening the public sector workforce | 36 | 6 | 10 | 9 | 5 | 6 | 4 | 2 | 1 | 1 |
| Areas of disparity, such as vaccination rates among the urban poor | 12 | 29 | 13 | 7 | 8 | 3 | 2 | 4 | 2 | 0 |
| Assisting state government initiatives for vaccine delivery | 4 | 10 | 15 | 7 | 15 | 10 | 10 | 6 | 3 | 0 |
| Strengthening advocacy efforts around newer vaccines/vaccine introduction | 2 | 4 | 5 | 11 | 16 | 10 | 12 | 8 | 10 | 2 |
| Improving vaccination coverage and research opportunities | 9 | 17 | 21 | 13 | 6 | 7 | 5 | 1 | 1 | 0 |
| Communications on vaccine use and addressing vaccine hesitancy | 6 | 6 | 6 | 10 | 10 | 13 | 18 | 8 | 2 | 1 |
| Research focused on reaching 'Zero Dose' children | 5 | 1 | 2 | 9 | 7 | 10 | 22 | 15 | 8 | 1 |
| Role of vaccines in preventing other healthcare outcomes (eg: Antimicrobial resistance) | 2 | 1 | 5 | 5 | 3 | 5 | 3 | 28 | 25 | 3 |
| Enhancing public private partnerships for vaccine delivery | 3 | 4 | 3 | 7 | 8 | 10 | 4 | 7 | 28 | 6 |

| **Sub-domain (n = 80)** | **Mean Weighted Rank** |
| --- | --- |
| Strengthening the public sector workforce | 8.03 |
| Areas of disparity, such as vaccination rates among the urban poor | 7.76 |
| Assisting state government initiatives for vaccine delivery | 6.29 |
| Strengthening advocacy efforts around newer vaccines/vaccine introduction | 5.16 |
| Improving vaccination coverage and research opportunities | 7.48 |
| Communications on vaccine use and addressing vaccine hesitancy | 5.73 |
| Research focused on reaching 'Zero Dose' children | 4.75 |
| Role of vaccines in preventing other healthcare outcomes (eg: Antimicrobial resistance) | 3.7 |
| Enhancing public private partnerships for vaccine delivery | 4.2 |

**Outbreak Preparedness**

| **N = 79** | **Rankings** |  |  |  |  |  |  |
| --- | --- | --- | --- | --- | --- | --- | --- |
| **Sub-domain** | **1** | **2** | **3** | **4** | **5** | **6** | **7** |
| Developing lab capacity and training | 21 | 10 | 15 | 11 | 18 | 4 | 0 |
| Enhancing public health surveillance networks | 22 | 26 | 16 | 10 | 5 | 0 | 0 |
| Conducting COVID-19 research for Maternal and Child Health sub-populations | 12 | 5 | 10 | 21 | 13 | 15 | 3 |
| Assisting state government initiatives for disease surveillance | 5 | 14 | 18 | 15 | 15 | 11 | 1 |
| Strengthening community knowledge and awareness of disease transmission | 9 | 12 | 14 | 6 | 17 | 18 | 3 |
| Enhancing infrastructure needs for disease management (eg: Oxygen, Modeling for healthcare service needs) | 7 | 7 | 6 | 15 | 10 | 30 | 4 |

| **Sub-domain (n = 79)** | **Mean Weighted Rank** |
| --- | --- |
| Developing lab capacity and training | 4.91 |
| Enhancing public health surveillance networks | 5.63 |
| Conducting COVID-19 research for Maternal and Child Health sub-populations | 1.91 |
| Assisting state government initiatives for disease surveillance | 4.27 |
| Strengthening community knowledge and awareness of disease transmission | 4.04 |
| Enhancing infrastructure needs for disease management (eg: Oxygen, Modeling for healthcare service needs) | 3.48 |

**Primary Care Integration**

| **N = 76** | **Rankings** |  |  |  |  |  |
| --- | --- | --- | --- | --- | --- | --- |
| **Sub-domain** | **1** | **2** | **3** | **4** | **5** | **6** |
| Nutrition education support through community workers | 23 | 14 | 20 | 11 | 7 | 1 |
| Supplemental nutrition delivery and effectiveness | 10 | 29 | 17 | 10 | 8 | 2 |
| Growth monitoring through regular check ups | 17 | 12 | 23 | 14 | 9 | 1 |
| Early childhood care or preschool | 8 | 12 | 12 | 32 | 10 | 2 |
| Supporting research around community service delivery and effectiveness | 15 | 7 | 3 | 6 | 41 | 4 |

| **Sub-domain (n = 76)** | Mean Weighted Rank |
| --- | --- |
| Nutrition education support through community workers | 4.42 |
| Supplemental nutrition delivery and effectiveness | 4.22 |
| Growth monitoring through regular check ups | 4.14 |
| Early childhood care or preschool | 3.61 |
| Supporting research around community service delivery and effectiveness | 3.17 |

**Maternal Health**

| **N = 76** | **Rankings** |  |  |  |  |  |  |  |
| --- | --- | --- | --- | --- | --- | --- | --- | --- |
| **Sub-domain** | **1** | **2** | **3** | **4** | **5** | **6** | **7** | **8** |
| Improving maternal immunization coverage | 15 | 12 | 10 | 12 | 12 | 6 | 5 | 4 |
| Encouraging at least 4-8 antenatal visits during pregnancy | 19 | 27 | 12 | 11 | 2 | 4 | 1 | 0 |
| Improving facility based safe delivery outcomes | 14 | 11 | 25 | 13 | 8 | 2 | 3 | 0 |
| Financial assistance/expenditures for births at an institution | 5 | 2 | 6 | 16 | 17 | 12 | 15 | 3 |
| Incentivizing use of Mother and Child Protection Cards | 5 | 5 | 1 | 4 | 16 | 25 | 17 | 3 |
| Improving maternal nutrition/supplementation (iron, folic acid supplementation) | 9 | 12 | 11 | 8 | 9 | 17 | 7 | 3 |
| Delivering family planning interventions at the time of childbirth | 3 | 5 | 8 | 9 | 11 | 10 | 27 | 3 |

| **Sub-domain (n = 76)** | **Mean Weighted Rank** |
| --- | --- |
| Improving maternal immunization coverage | 5.32 |
| Encouraging at least 4-8 antenatal visits during pregnancy | 6.45 |
| Improving facility based safe delivery outcomes | 5.89 |
| Financial assistance/expenditures for births at an institution | 4.04 |
| Incentivizing use of Mother and Child Protection Cards | 3.64 |
| Improving maternal nutrition/supplementation (iron, folic acid supplementation) | 4.82 |
| Delivering family planning interventions at the time of childbirth | 3.72 |

**Neonatal Health**

| **N = 73** | **Rankings** |  |  |  |  |  |  |  |  |
| --- | --- | --- | --- | --- | --- | --- | --- | --- | --- |
| **Sub-domain** | **1** | **2** | **3** | **4** | **5** | **6** | **7** | **8** | **9** |
| Neonatal resuscitation to reduce perinatal asphyxia | 30 | 15 | 9 | 7 | 3 | 4 | 3 | 2 | 0 |
| Improving initiation of breast feeding in hospital | 9 | 16 | 20 | 9 | 7 | 4 | 5 | 3 | 0 |
| Prevention and management of newborn sepsis | 3 | 16 | 14 | 13 | 9 | 9 | 8 | 1 | 0 |
| Kangaroo mother care for low birth weight babies | 1 | 4 | 10 | 22 | 25 | 4 | 4 | 3 | 0 |
| Improving quality of care during labor and birth | 22 | 6 | 11 | 9 | 13 | 11 | 0 | 1 | 0 |
| Community-based extra care for preterm/low birthweight babies | 4 | 4 | 5 | 8 | 5 | 20 | 20 | 6 | 1 |
| Vaccinating the neonate prior to discharge from the facility | 0 | 1 | 0 | 3 | 3 | 13 | 23 | 26 | 4 |
| Enhancing early home based newborn care | 3 | 9 | 3 | 1 | 6 | 8 | 9 | 30 | 4 |

| **Sub-domain (n = 73)** | **Mean Weighted Rank** |
| --- | --- |
| Neonatal resuscitation to reduce perinatal asphyxia | 7.38 |
| Improving initiation of breast feeding in hospital | 6.51 |
| Prevention and management of newborn sepsis | 6 |
| Kangaroo mother care for low birth weight babies | 5.51 |
| Improving quality of care during labor and birth | 6.68 |
| Community-based extra care for preterm/low birthweight babies | 4.51 |
| Vaccinating the neonate prior to discharge from the facility | 2.99 |
| Enhancing early home based newborn care | 3.82 |

**Infectious Diseases**

| **n = 77** | **Ranks** |  |  |  |  |  |  |  |  |
| --- | --- | --- | --- | --- | --- | --- | --- | --- | --- |
| **Sub-domain** | **1** | **2** | **3** | **4** | **5** | **6** | **7** | **8** | **9** |
| Dengue prevention and management | 15 | 10 | 15 | 11 | 10 | 10 | 3 | 3 | 0 |
| COVID-19 | 20 | 11 | 12 | 10 | 10 | 3 | 6 | 2 | 3 |
| Pediatric and maternal screening and treatment for tuberculosis | 11 | 19 | 13 | 11 | 13 | 6 | 2 | 2 | 0 |
| Maternal and neonatal screening for HIV | 6 | 7 | 11 | 14 | 15 | 15 | 6 | 3 | 0 |
| Pediatric and maternal screening and treatment for malaria | 2 | 7 | 6 | 8 | 13 | 22 | 17 | 2 | 0 |
| Pediatric and maternal screening for influenza and other viral illnesses | 3 | 0 | 6 | 7 | 4 | 13 | 19 | 23 | 2 |
| Diarrhoeal diseases prevention and treatment | 14 | 17 | 7 | 12 | 6 | 6 | 11 | 4 | 0 |
| Neglected tropical diseases (Hookworm, Dengue, Rabies, Ascariasis) | 2 | 4 | 5 | 3 | 5 | 2 | 12 | 37 | 7 |

| **Sub-domain (n = 77)** | **Mean Weighted Rank** |
| --- | --- |
| Dengue prevention and management | 6.38 |
| COVID-19 | 6.48 |
| Pediatric and maternal screening and treatment for tuberculosis | 6.58 |
| Maternal and neonatal screening for HIV | 5.58 |
| Pediatric and maternal screening and treatment for malaria | 4.83 |
| Pediatric and maternal screening for influenza and other viral illnesses | 3.74 |
| Diarrhoeal diseases prevention and treatment | 6.21 |
| Neglected tropical diseases (Hookworm, Dengue, Rabies, Ascariasis) | 3.29 |

1. **“Other” Write-In Answers (Table 1. Likert/Ranking. Table 2. Overall (Q9, etc))**

**Vaccine Preventable Diseases**

Vaccine Preventable Diseases

| **Likert "Other"** | **Ranking "Other"** |
| --- | --- |
| **Adverse Events (n = 4)** | **Adverse Events (n = 3)** |
| Adverse effects following Immunization (n = 1) | AEFI (n = 1) |
| Adverse Events from Vaccination (n = 1) | AEFI, Vaccine utilization pattern , Immunization waste management ,immunization status among the migratory population especially in Urban area (n = 1) |
| AEFI, Vaccine utilization pattern, Immunization waste management, immunization status among the migratory population especially in Urban area (n = 1) | Studying adverse reactions (n = 1) |
| Studying vaccine adverse reactions (n = 1) | **Surveillance (n = 2)** |
| **Vaccine Storage (n = 4)** | Recording and tracing (n = 1) |
| Cold chain and efficient and affordable logistic (n = 1) | Surveillance on vaccine preventable diseases to setup priorities (n = 1) |
| Maintaining cold chain and appropriate training (n = 1) | **Evaluation of Immunization Programs (n = 2)** |
| Proper storage of vaccine (n = 1) | independent evaluations with effectiveness, impact etc academic institutions (n = 1) |
| **Surveillance (n = 5)** | Implementation research; Ongoing evaluation of immunization programmes (n = 1) |
| Real time data reporting within public health system (n = 1) |  |
| Recording and tracing (n = 1) |  |
| Serosurveillance for VPD (n = 1) |  |
| Surveillance for Vaccine Preventable Diseases (n = 1) |  |
| Surveillance on vaccine preventable diseases to setup priorities (n = 1) |  |
| Other |  |
| vaccine trial acceleration by collaboration (n = 1) |  |
| Paucity of Human resource (n = 1) |  |
| Outreach to lesser reached populations (migrants etc) (n=3) |  |
|  |  |

| **Likert "Other"** | **27** | **Ranking "Other"** | **23** |
| --- | --- | --- | --- |
| Adverse effects following Immunization (n = 1) | 1 | independent evaluations with effectiveness, impact etc academic institutions (n = 1) | 1 |
| Adverse Events from Vaccination (n = 1) | 1 | AEFI (n = 1) | 1 |
| AEFI, Vaccine utilization pattern , Immunization waste management ,immunization status among the migratory population especially in Urban area (n = 1) | 1 | AEFI, Vaccine utilization pattern , Immunization waste management ,immunization status among the migratory population especially in Urban area | 1 |
| Affordability (n = 1) | 1 | Affordability | 1 |
| Behavioral Aspects in Vaccine Administration and Vaccine uptake by beneficiaries (n = 1) | 1 | Behavioral Aspects in Vaccine Administration and Vaccine uptake by beneficiaries | 1 |
| Cold chain and efficient and affordable logistic (n = 1) | 1 | Cold chain and logistics | 1 |
| Community System Mobilization (n = 1) | 1 | Community System Mobilization | 1 |
| Establishing Vaccination Networks for better understanding and Dissemination of Information and conducting Research (n = 1) | 1 | disparity between actual performance and declared statistics | 1 |
| Implementation research, ongoing evaluation (n = 1) | 1 | Establishing Vaccination Networks for better understanding and Dissemination of Information and conducting Research | 1 |
| Maintaining cold chain and appropriate training (n = 1) | 1 | Factors hindering immunization coverage | 1 |
| Making the family own the responsibility (n = 1) | 1 | Implementation research; Ongoing evaluation of immunization programs | 1 |
| Myths and misconceptions influencing vaccine utilization (n = 1) | 1 | Improving vaccination among migrant workers | 1 |
| Paucity of Human resource (n = 1) | 1 | Making the family own the responsibility | 1 |
| Proper storage of vaccine (n = 1) | 1 | Myths and misconceptions influencing vaccine utilization | 1 |
| Real time data reporting within public health system (n = 1) | 1 | Paucity of public sector Human Resource | 1 |
| Recording and tracing (n = 1) | 1 | Real time data management | 1 |
| research on coverage esp. of marginalized children (n = 1) | 1 | Recording and tracing | 1 |
| Research on route of administration of vaccination on mass scale (n = 1) | 1 | Research on infectious diseases which may need to be prevented by vaccines in future eg.COVID 19 and other future pandemics | 1 |
| research to understand reasons for poor vaccine delivery (n = 1) | 1 | Research on route of administration of vaccination on mass scale | 1 |
| Sero-surveillance for VPD (n = 1) | 1 | Strong R&D mechanism | 1 |
| Share proper information in public domain (n = 1) | 1 | Studying adverse reactions | 1 |
| Studying vaccine adverse reactions (n = 1) | 1 | Surveillance on vaccine preventable diseases to setup priorities | 1 |
| Surveillance for Vaccine Preventable Diseases (n = 1) | 1 | vaccine trial acceleration by collaboration | 1 |
| Surveillance on vaccine preventable diseases to setup priorities (n = 1) | 1 |  | 1 |
| Transportation access (n = 1) | 1 |  |  |
| Vaccination among migrants (Temporary migrants working at construction sites)) (n = 1) | 1 |  |  |
| vaccine trial acceleration by collaboration (n = 1) | 1 |  |  |

| **Adverse Events** | 5 |
| --- | --- |
| Adverse event surveillance, procedures for informed consent for vaccination in all populations (lacking), monitoring cold chain | 1 |
| AEFI surveillance | 1 |
| post-vaccination adverse events and concerns of parents/mothers. | 1 |
| Reactions following the delivery of a vaccine, I think is important. | 1 |
| ROLE OF ADVERSE EFFECTS FOLLOWING IMMUNIZATION AND ITS REPORTING | 1 |
| **Affordability** | 2 |
| 1.Develop locally relevant cost‑effective strategies to expand the coverage of UIP  2.Improving administrative data quality | 1 |
| **Immunization Systems** | 11 |
| Consideration for making vaccines available daily, in public centres, instead of once a month or fortnight. | 1 |
| Adequate and appropriate support to ANMs and ASHAs in conducting Out reach sessions( village/ slum) | 1 |
| Antibody testing | 1 |
| Emphasis on booster doses of vaccines for vaccine preventable diseases in mid school age group | 1 |
| Engagement of NGOs or grass root level organization in implementation of vaccination specially in rural areas and marginalized sectors. | 1 |
| Establishing Vaccination Networks for better understanding and Dissemination of Information and conducting Research and capacity building in the field of vaccine preventable diseases | 1 |
| Exploring how newer vaccines can be incorporated in RI | 1 |
| in-depth understanding of health workers about missed and delayed vaccination | 1 |
| Increasing public health outlay For capacity building in public health initiation of public health teaching-learning More funding to encourage public health translational research | 1 |
| Research on newer vaccines, passive therapy | 1 |
| Research on repurposing already existing vaccine. | 1 |
|  |  |
| **Cold Chains and Vaccine Distribution** | 8 |
| Supply chain management& capacity building | 1 |
| Adverse event surveillance, procedures for informed consent for vaccination in all populations (lacking), monitoring cold chain | 1 |
| Availability and maintenance of supply chain | 1 |
| Home delivery the vaccine for preventable disease | 1 |
| Regular uninterrupted supply of vaccines | 1 |
| Training of healthcare workforce for vaccine administration and storage/ transport to outreach areas. | 1 |
| vaccination during emergencies and cold chain | 1 |
| Vaccine to marginalized population should prioritized. All kinds of marginalization whether geographic, economic or social. | 1 |
| **Surveillance and Evaluation Systems** | 12 |
| Adverse event surveillance, procedures for informed consent for vaccination in all populations (lacking), monitoring cold chain | 1 |
| Data reporting | 1 |
| Mapping and tracking of children once they are born till their full immunization is complete with help of Information technology or GIS mapping or other technological solutions | 1 |
| Occurrence of VPD among fully vaccinated children | 1 |
| Reemergence of Vaccine preventable diseases with the waning immunity or neglect of immunization as in Diphtheria. | 1 |
| Sero-surveillance for VPD | 1 |
| strengthening the follow-up mechanism of dropouts and left-outs through area-specific app-based tracking.  AEFI Immunization among migratory population | 1 |
| Surveillance giving priority to Laboratory Diagnosis, Improving OPD surveillance especially in major Hospitals both in the Public & Private Sector. Typhoid vaccine universalization. Role of Campaign approaches (Indra-Dhanush etc) VS Promoting Routine Immunization. | 1 |
| Surveillance including involvement of private sector | 1 |
| Surveillance of Vaccine preventable Diseases | 1 |
| Surveillance on vaccine preventable diseases to setup priorities | 1 |
| Timely receipt of vaccine as per WHO guidelines (keeping the gaps intact) | 1 |
| **Behavioral Aspects** | 4 |
| How optimal nutrition in the initial years (exclusive breastfeeding and continued breastfeeding) is the most effective vaccine to prevent diseases? How to support mothers in this? | 1 |
| Public awareness and sensitization to necessity for vaccination | 1 |
| Role of local self Government, community groups and civil society organizations in reducing stigma and behavior change of the community around vaccination and improving vaccine coverage | 1 |
| The role of nutrition, WASH which will boost immunity to prevent vaccine preventable diseases in future. | 1 |
| **Novel research** | 2 |
| Research to develop vaccines for diseases prevalent in India. | 1 |
| COVID-19 | 1 |
|  |  |

Outbreak Preparedness

| **Likert "Other"** | **Ranking "Other"** |
| --- | --- |
| **Social Determinants of Health (n = 2)** | **Infection Prevention (n = 2)** |
| Reasons of disparity in spread in different regions and how to stop rapid spread to vulnerable populations (n = 1) | Infection Prevention & Control in Facility & Community (n = 1) |
| Impact of the outbreak on the social determinants of health (n = 1) | Prevention of spread (n = 1) |
| **Service Delivery (n = 2)** | **Training Staff (n = 7)** |
| Enhancing service delivery Network and Strengthening Reporting of data from the Generation points (n = 1) | Training of disaster management teams and ensure rapid deployment (n = 1) |
| Ensuring access and service delivery in lockdown like periods (n = 1) | Trained manpower (n = 1) |
| **Healthcare Preparation (n = 6)** | preparedness of health staffs (n = 1) |
| Increase the facility of health care personnel (n = 2) | training grass root level workers in appropriate vaccine practices (n = 1) |
| Capacity building of health workers, health facilities (n = 2) | Capacity building of health workers (n = 1) |
| Strengthening/ Training and re-training health workers (n = 2) | State preparedness: Simulation exercises, measures to increase bed capacity, HR preparedness, roles of various stakeholders, etc. (n = 1) Under public health surveillance: scope of GIS in mapping of cases, linkage to public health facilities. |
| Covid related | Training & Retraining of health workers (n = 1) |
| long term effects of COVID-19 among pregnant women | **Service Delivery (n = 2)** |
| Effect on non-covid services | Ensuring access and service delivery in lockdown like periods (n = 1) |
| Exploring vertical transmission | Enhancing service delivery Network and Strengthening Reporting of data from the Generation points (n = 1) |
| Birth Outcomes among Pregnant women with COVID | **Increase Staff (n =2)** |
|  | Encouraging staff to stay in rural health centres (n = 1) |
|  | Increase the Primary health care workers (n = 1) |
|  | **Surveillance (n = 2)** |
|  | Surveillance in OPDs of Major Hospitals (n = 1) |
|  | reviewing surveillance data for action (n = 1) |

| **Likert "Other"** **(n = 24)** | **24** | **Ranking "Other" (n = 29)** | **29** |
| --- | --- | --- | --- |
| Funding | 1 | Encouraging staff to stay in rural health centres | 1 |
| Community engagement in disease outbreak preparation | 1 | Developing Early warning signals | 1 |
| Maximum test number | 1 | Infection Prevention & Control in Facility & Community | 1 |
| Reasons of disparity in spread in different regions and how to stop rapid spread to vulnerable populations (n = 1) | 1 | Training of disaster management teams and ensure rapid deployment | 1 |
| Making the target population capable of utilizing public health services | 1 | close follow up | 1 |
| Central and State government co-ordination | 1 | prevention of spread | 1 |
| NGOs involvement | 1 | Trained manpower | 1 |
| Trained manpower | 1 | preparedness of health staffs | 1 |
| long term effects of COVID-19 among pregnant women | 1 | Specific anti viral drug and vaccine | 1 |
| Improve attitudes and motivation levels of public health workers | 1 | using arts and local knowledge for communicating | 1 |
| Exploring vertical transmission | 1 | vaccine research | 1 |
| Increase the facility of health care personnel | 1 | any other | 1 |
| Capacity building of health workers | 1 | Exploring vertical transmission | 1 |
| Surveillance in OPDs of Major Hospitals | 1 | Improving other determinants of health | 1 |
| Conducting COVID-19 research for sequalae among mother and children | 1 | Increase the Primary health care workers | 1 |
| Social Behaviour Change Approaches to prevent COVID infection spread | 1 | Data for Decisions | 1 |
| effect on non covid services | 1 | training grass root level workers in appropriate vaccine practices | 1 |
| Enhancing service delivery Network and Strengthening Reporting of data from the Generation points | 1 | Capacity building of health workers | 1 |
| Strengthening/ Training and re-training health workers | 1 | Surveillance in OPDs of Major Hospitals | 1 |
| Inter departmental convergence for institutionalization | 1 | reviewing surveillance data for action | 1 |
| Ensuring the infrastructure and logistics at each place to prevent Covid-19 Infection | 1 | Improving biomedical research capacity | 1 |
| Impact of the outbreak on the social determinants of health | 1 | Social Behaviour Change Approaches to prevent COVID infection spread | 1 |
| Birth Outcomes among Pregnant women with COVID | 1 | State preparedness: Simulation exercises, measures to increase bed capacity, HR preparedness, roles of various stakeholders, etc. Under public health surveillance: scope of GIS in mapping of cases, linkage to public health facilities. | 1 |
| Ensuring access and service delivery in lockdown like periods | 1 | supply chain management | 1 |
|  |  | Enhancing service delivery Network and Strengthening Reporting of data from the Generation points | 1 |
|  |  | Training & Retraining of health workers | 1 |
|  |  | Inter departmental convergence for institutionalization | 1 |
|  |  | Impact of the outbreak on the social determinants of health | 1 |
|  |  | Ensuring access and service delivery in lockdown like periods | 1 |

| **COVID19 Specific** | 4 |
| --- | --- |
| Challenges in early initiation of Breast feeding among babies born to COVID positive mothers. Infection rate among babies born to COVID positive mothers. | 1 |
| focus on stigma and discrimination involved with COVID-19 and its management procedures, how to rethink antenatal and postnatal care plans in the days of outbreaks and containment like using tele-consults etc | 1 |
| The preparedness of the population after the covid experience | 1 |
| To objectively assess vulnerability of children for Covid 19, clinical profile and their possible role in community transmission of the disease. | 1 |
| **Role of Public Health and Healthcare Workers** | 9 |
| Strengthening primary healthcare infrastructure network  Encouragement of primary healthcare providers by cash, kind and avenues of growth | 2 |
| challenges of frontline workers during outbreaks | 1 |
| Health systems readiness. | 1 |
| Increase the Primary health care worker and their facilities | 1 |
| Exclusive Call centres or 'priority desks' in existing call Centres to provide Information and guidance to Mothers/ families | 1 |
| Evidence base for neonatal testing strategy | 1 |
| Aetiology | 1 |
| Destigmatising the disease | 1 |
| **Novel research** | 2 |
| Research on relation of viral infection with co-morbidity. | 1 |
| Zoonotic diseases | 1 |
| **Outbreak Management** | 12 |
| Data management | 1 |
| Enhancing service delivery Network and Strengthening Reporting of data from the Generation points | 1 |
| Gaps in training in epidemiology, roles and responsibilities of epidemiologists | 1 |
| Operational research on the ability of people (health workforce) who have the capacity to carry out outbreak investigation and management might be an interesting area. | 1 |
| Outbreak investigations, management, and Final Reporting Firming Accountability For surveillance in specialized units of Major Hospitals (e.g Diphtheria-ENT,) | 1 |
| Preparedness for uninterrupted maternal and child health services and studying factors which led to disturbance of routine maternal and child health services early in the course of pandemic | 1 |
| State preparedness: Simulation exercises, measures to increase bed capacity, HR preparedness, roles of various stakeholders, etc. Under public health surveillance: scope of GIS in mapping if cases, linkage to public health facilities. | 1 |
| Train policy makers for mange pandemics based on scientific facts and not encourage political interference | 1 |
| what works in outbreak preparedness | 1 |
| Research on effectiveness of interventions | 1 |
| Participatory research for community-based solutions for improving access and utilization of existing flagship govt. programmes to prevent disease transmission,  Action research on strength of the approaches like  - capacity building of the front line workers to prevent malnutrition,  - improving utilization of primary health care services to prevent illness,  - preventing social barriers like child labour, early marriages and school drop-outs through convergence of programs and multi-stakeholder engagement as well as  - increasing participation of children, adolescents and women in outbreak preparedness and prevention | 1 |
| participatory research on pandemic preparedness with global research consortium. This is a new area and not much evidence has been generated especially in LMIC and disadvantaged population ,, this is with regard to information/ misinformation sharing and developing community resilience, a and co developing operational frameworks through rigorous research models and modelling | 1 |
| **Rural/Poor Outreach** | 3 |
| wide publicity in rural areas on ' How. where to get MCH services in the times of pandemic | 1 |
| Affordability and free distribution for poor people and easy accessibility | 1 |
| Encouraging staff to stay in rural health centres | 1 |

Primary Care Integration

| **Likert "Other"** | **Ranking "Other"** |
| --- | --- |
| **Nutrition (n = 9)** | **Nutrition (n = 4)** |
| Micronutrients (n = 1) | nutritional intervention (n =1) |
| Ensuring nutrition for pregnant mothers (n = 1) | Preparation fo Supplementary nutrition items using local recipes (n = 1) |
| Preparation of supplementary nutrition items using local recipes (n = 1) | Care of SAM and MAM at Nutrition rehabilitation Centres (NRCs)/ Facilities and at Home (n = 1) |
| Research on Sustaining nutritional support in community and studying changing trends of nutrition (n = 1) | maternal nutrition (n = 1) |
| System strengthening for delivery of nutrition programs (n = 1) | **Community Health Workers (n = 5)** |
| maternal nutrition (n = 1) | Primary Health care workers continuation of job (n = 1) |
| Community ownership towards nutrition of Children below 5 (n = 1) | Ashas are overburdened and underpaid. Need to strengthen and motivate the workforce (n = 1) |
| Nutritive assessment of quality of meals served at the anganwadis to under 5 children (n = 1) | integration of activities of ASHAs and Anganwadi workers (n = 1) |
| Care of SAM and MAM at Nutrition rehabilitation Centres (NRCs)/ Facilities and at Home (n = 1) | Improving infrastructure at PHCs (n = 1) |
| **ASHA and AWC (n = 2)** | Involvement of Institution working in the field (n = 1) |
| Supervise and maintain ongoing services normally provided by ASHA and AWW without disruption at all times (n = 1) |  |
| Engagement of family and parents in ASHA and AWC activities (n = 1) |  |
| **Other systems strengthening (5)** |  |
| Tribal outreach |  |
| Audit, surveillance, guidelines |  |
| Strengthening Antenatal Care Delivery, Use of technology for real-time data record |  |
| **Novel research** |  |
| use of AI for effective services of ICDS |  |

| **Likert "Other"** | **22** | **Ranking "Other"** | **29** |
| --- | --- | --- | --- |
| Micronutrients | 1 | Sanitation | 1 |
| Supervise and maintain ongoing services normally provided by ASHA and AWW without disruption at all times | 1 | Ashas are overburdened and underpaid. Need to strengthen and motivate the workforce | 1 |
| Targeting specific groups like tribal community of Jungle Mahal, Sundarban area | 1 | Robust infrastructure development for delivery | 1 |
| proper knowledge & practice of newborn care | 1 | Implementation | 1 |
| Effect of one administrative head for both | 1 | Early access and utilization of treating common childhood ailments | 1 |
| designing operational framework and guidelines for LMICs | 1 | nutritional intervention | 1 |
| Ensuring nutrition for pregnant mothers | 1 | NGOs engagement | 1 |
| Both the Workers shall work in convergence as outcome is same | 1 | Evaluation of Poshan aviyaan | 1 |
| Use of AI for effective services of ICDS | 1 | Designated nodal person | 1 |
| Preparation of supplementary nutrition items using local recipes | 1 | Effect of one administrative head | 1 |
| improving maternal death audit | 1 | Designing operational framework and guidelines for LMICs | 1 |
| Building social norms for better MCH | 1 | UHC for child health issues | 1 |
| Research on Sustaining nutritional support in community and studying changing trends of nutrition | 1 | Both the Workers shall work in convergence as outcome is same | 1 |
| Growth Surveillance | 1 | workload mapping | 1 |
| System strengthening for delivery of nutrition programs | 1 | any other | 1 |
| maternal nutrition | 1 | Exclusive breastfeeding | 1 |
| Involvement of Education Institute working in the field at all levels | 1 | Primary Health care workers continuation of job | 1 |
| Strengthening Antenatal Care Delivery, Use of technology for real-time data record | 1 | out of pocket expenditure to meet health needs | 1 |
| Community ownership towards nutrition of Children below 5 | 1 | Real time reporting | 1 |
| Engagement of family and parents in ASHA and AWC activities | 1 | Understanding what is the new normal in India | 1 |
| Nutritive assessment of quality of meals served at the anganwadis to under 5 children | 1 | Preparation of Supplementary nutrition items using local recipes | 1 |
| Care of SAM and MAM at Nutrition rehabilitation Centres (NRCs)/ Facilities and at Home | 1 | severe acute maternal morbidities and near‑miss events | 1 |
|  |  | Building social norms for better MCH | 1 |
|  |  | maternal nutrition | 1 |
|  |  | integration of activities of ASHAs and Anganwadi workers | 1 |
|  |  | Improving infrastructure at PHCs | 1 |
|  |  | Involvement of Institution working in the field | 1 |
|  |  | Engagement of family and parents in ASHA and AWC activities | 1 |
|  |  | Care of SAM and MAM at Nutrition rehabilitation Centres (NRCs)/ Facilities and at Home | 1 |

| **Community** | **17** |
| --- | --- |
| - Community participation for recognizing malnutrition as an important issue in the life cycle of a human being,  - involvement of local women/children groups to support front-line workers in identifying and tracking of drop-outs from the public health and nutrition services,  - Early identification of nutrition-risk mothers, including obesity - identification of workable strategies for primary health care integration through community involvement - adolescent sexual reproductive health issues | 1 |
| Accessibility of community to ASHA workers and Anganwadi workers in case of any sickness of children not routinely discussed with them | 1 |
| Asha workers continuation of jobs. | 1 |
| Minimizing over-dependency on community-level workers & strengthening Community Health & welfare centers | 1 |
| Operationalization of Health & Wellness Centres (Suswasthya Kendra) for providing comprehensive primary health care | 1 |
| Research for health system strengthening of primary health care in terms of human resources, their training and logistics | 1 |
| The workload mapping of frontline workers' work and rethinking tasks assigned so they focus on community engagement rather than health worker support | 1 |
| There are many areas in this space - Behaviour change communication related research, barriers to effective service delivery, attitudes and perception of the community towards primary health care workers and so on.... | 1 |
| Up-gradation/Modification of Primary Health Center model | 1 |
| WASH Activities at family, household and community level. | 1 |
| research on community participation in nutritional rehabilitation | 1 |
| Sanitation | 1 |
| Access to benefits | 1 |
| Involvement of Education Institute Like Medical Colleges, Nutrition College, Nursing Schools etc. working in the field at all levels | 1 |
| Mobilizing sufficient fund and resources | 1 |
| Parenting skill development | 1 |
| Targeting specific vulnerable group like Tribal people of Jungle Mahal and Sundarban area. | 1 |
| **Nutrition** | **3** |
| Nutrition education at all curriculum | **1** |
| Adolescent nutrition needs | 1 |
| Timely and adequate complementary feeding in terms of quality and quantity | 1 |
| **Early Life** | **4** |
| 1 Mechanisms to track and support families, to prevent 'Slipping back' of recovered children in to SAM & MAM 2.More emphasis on ' Stunting'- measuring skills of FLWs, sensitization and Nutrition education to Families and on relevant research covering special food items and Recommended daily intakes etc 3.Dessimination of the concept of '1st 1000 days' in improvised methods and techniques to Rural, Illiterate and Poverty ridden Young parents. | 1 |
| Availability of treatment facilities at primary health care facilities. Proper care of mother and child during the first 1000 days of life. | 1 |
| Care for children between 6 months to 3 years old. | 1 |
| awareness regarding early childhood early childhood infections/ inborn errors/ PID | 1 |
| **Technology/ Novel research** | **4** |
| 1.Development and validation of low‑cost technologies for screening, referral and management of childhood pneumonia and ARI  2.Strategies to promote WASH practices in the community to improve child health | 1 |
| Decision making tool to have access to granular data. Ability of policy maker to use technology to improve health outcome. AI in maternal health. | 1 |
| R&D for artificial intelligence tools for more effective growth monitoring, identification of nutritional deficiencies, designing nutritional supplement based on individual needs (customized) | 1 |
| Use of real-time data capturing methods in public health | 1 |
| **Behavioral** | **2** |
| Treatment compliance | 1 |
| There are many areas in this space - Behaviour change communication related research, barriers to effective service delivery, attitudes and perception of the community towards primary health care workers and so on.... | 1 |
| **Resources** | **3** |
| Access to benefits | 1 |
| Involvement of Education Institute Like Medical Colleges, Nutrition College, Nursing Schools etc. working in the field at all levels | 1 |
| Mobilizing sufficient fund and resources | 1 |
| **Epidemiologic Integrations** | 3 |
| Doing research to understand what is the normal in Indian setting e.g.Weight of the pregnant women | 1 |
| Life course epidemiological studies and social determinants of child health and addressing them | 1 |
| Reasons for occurrence of low birth weight babies | 1 |

Maternal Health

| **Likert "Other"** | **Ranking "Other"** |
| --- | --- |
| **Identification of High-Risk Pregnancies (n = 3)** | **Post-natal Care (n = 3)** |
| Predictors of unfavorable outcomes in pregnancy (n = 1) | Improved post-natal care (n = 1) |
| Effectiveness of VHSND platform in identifying and referring high risk pregnant women and Malnourished children (n = 1) | Improvement in post-natal visits (n = 1) |
| timely identification and follow-up care to High Risk pregnancies(HRPs) (n = 1) | Birth weight monitoring (n = 1) |
| **Systems strengthening (7)** | **High Risk Pregnancies (n = 6)** |
| Quality of ANC,INC and PNC and Home based maternal care | Identifying High Risk in third trimester (n = 1) |
| Availability of cash transfer during pregnancy and early childhood | High risk screening in ANC (n = 1) |
| Improvement in post-natal visits | Predictors of unfavorable outcomes in pregnancy (n = 1) |
| Provision of C-Section facility on the prescribed norm. | Effectiveness of VHSND platforms in identifying and referring high risk pregnant women and Malnourished children (n = 1) |
| timely identification and follow-up care to High Risk pregnancies(HRPs) | Identification of HRP, preparedness of FRUs and triage (n = 1) |
| Involvement of Private Sector for Improving the strategies | identification and care of HRPs (n = 1) |
| Effectiveness of VHSND platform in identifying and referring high risk pregnant women and Malnourished children | **Antenatal Care (n = 4)** |
| **Novel research** | Communication with mother before birth of child (n = 1) |
| implementation research on designing and evaluating innovations and tele mentoring and digital platforms for capacity building | Quality ANC,INC and PNC (n = 1) |
| Use of data capturing technologies | High risk screening in ANC (n = 1) |
|  | Adequate infrastructure for proper ANCs at SC-level (n = 1) |
|  | **Maternal Well-Being (n = 4)** |
|  | Self-care of mothers (n = 1) |
|  | Family counselling (n = 1) |
|  | Improving education level and preventing school drop-outs (n = 1) |
|  | Maternal mental health (n = 1) |

| **Likert "Other"** | **22** | **Ranking "Other"** | **35** |
| --- | --- | --- | --- |
| Pregnancy | 1 | Identifying High Risk in third trimester | 1 |
| Respectful Maternity Care | 1 | Improving education level and preventing school drop-outs | 1 |
| Quality of ANC,INC and PNC and Home based maternal care | 1 | Adolescence | 1 |
| Availability of cash transfer during pregnancy and early childhood | 1 | Respectful Maternity Care | 1 |
| Family counselling for care | 1 | Quality ANC, INC and PNC | 1 |
| Improvement in post-natal visits | 1 | High risk screening in ANC | 1 |
| Implementation research on designing and evaluating innovations and tele monitoring and digital platforms for capacity building | 1 | Self-care of mothers | 1 |
| Planning for pregnancy, childbirth and after and male involvement in each | 1 | Family counselling | 1 |
| Predictors of unfavorable outcomes in pregnancy | 1 | exploring the lacunae in service delivery | 1 |
| Provision of CSection facility on the prescribed norm. | 1 | puerperal period care of mother | 1 |
| Basic exercises for pregnant women | 1 | Adequate infrastructure for proper ANCs at SC-level | 1 |
| Adolescent programming for improving maternal outcomes | 1 | Improved post-natal care | 1 |
| Post-partum hemorrhage is still most prevalent cause of death due and needs to be addressed | 1 | Improvement in post-natal visits | 1 |
| IFA | 1 | implementation research on designing and evaluating innovations and tele mentoring and digital platforms for capacity building | 1 |
| Maternal Nutrition | 1 | improving male participation in pregnancy etc, and planning for pregnancy and after | 1 |
| treatment of anaemia at primary health care | 1 | Any other | 1 |
| Involvement of Private Sector for Improving the strategies | 1 | Predictors of unfavorable outcomes in pregnancy | 1 |
| Use of data capturing technologies | 1 | Maternal mental health | 1 |
| Community ownership towards health | 1 | Communication with mother before birth of child | 1 |
| Behavioral and cultural aspects | 1 | Birth weight monitoring | 1 |
| Effectiveness of VHSND platform in identifying and referring high risk pregnant women and Malnourished children | 1 | Provision of Chemo-oncology facility | 1 |
| timely identification and followup care to High Risk pregnancies(HRPs) | 1 | Basic exercises for Pregnant women | 1 |
|  |  | Adolescent programming for improving maternal outcomes | 1 |
|  |  | Post partum hemorrhage is still most prevalent cause of death due and needs to be addressed | 1 |
|  |  | Screening, diagnosis & management of Gestational Diabetes Mellitus (GDM) Abuse & violence (including intimate partner violence) during pregnancy Birth preparedness | 1 |
|  |  | treatment if anaemia at primary health care level | 1 |
|  |  | Anaemia management | 1 |
|  |  | Improving infrastructure | 1 |
|  |  | Involvement of Private Sector for Improving the strategies | 1 |
|  |  | Use of real-time data capturing techniques | 1 |
|  |  | community ownership towards Maternal health | 1 |
|  |  | Behavioral and cultural aspects | 1 |
|  |  | Identification of HRP, preparedness of FRUs and triage | 1 |
|  |  | Effectiveness of VHSND platforms in identifying and referring high risk pregnant women and Malnourished children | 1 |
|  |  | identification and care of HRPs | 1 |

| **Family Planning** | 5 |
| --- | --- |
| 1. Safe abortions for needy women/ girls in remote /rural areas 2 Freedom to the married women in- timing of pregnancy, and adoption of Family Planning | 1 |
| Barriers to family planning services in the community | 1 |
| Human Resource in Public & Private Sector for Maternal & Newborn Care Midwifery-led Obstetric & Newborn Care Quality of care- antenatal, intrapartum, postnatal | 1 |
| Awareness about and utilization of services under schemes like JSSY, PMSMA, JSY | 1 |
| Explore benefits of home delivery in the urban areas(where hygienic practices are feasible). Role of father in the maternal health, supportive system at home and work place that can improve the maternal and child health | 1 |
| **Transportation** | 2 |
| The movement of pregnant women in between the communities during the time of pregnancy (after conception going to her mother's place and coming to in-law's place once the baby becomes 4-5 months old). | 1 |
| Transportation of pregnant women for institutional delivery | 1 |
| **Antenatal Care** | 9 |
| Access to delivery services, maternal care homes, ante-natal care in the community, access to ultrasound scanning 3 times during pregnancy. | 1 |
| Antenatal care | 1 |
| Antenatal preparedness for breast feeding | 1 |
| Identifying High Risk in third trimester | 1 |
| IT based clinical decision support system and high-risk predictions | 1 |
| Human Resource in Public & Private Sector for Maternal & Newborn Care Midwifery-led Obstetric & Newborn Care Quality of care- antenatal, intrapartum, postnatal | 1 |
| Research targeting use of multivitamin supplementation in place of only iron + folic acid supplementation, and continued supplementation during lactation, for preventable nutrient-mediated teratogenesis in the offspring. | 1 |
| Screening, diagnosis & management of Gestational Diabetes Mellitus (GDM) Abuse & violence (including intimate partner violence) during pregnancy Birth preparedness | 1 |
| Maternal nutrition | 1 |
| **Maternal Morbidity Identification** | 3 |
| Availability and utility of emergency transport services  Maternal morbidity; identification and management | 1 |
| 1.Development and validation of algorithms for prevention, early detection and management of severe acute maternal morbidities  2.Strategies to improve quality of care during childbirth in the public health system 3.Early identification, referral and management of high‑risk pregnancies | 1 |
| reporting maternal deaths and near miss | 1 |
| **Women Empowerment** | 6 |
| Women empowerment in all fields of life from wage earners to army officers | 1 |
| - early marriage, early pregnancy - Couple engagement in family planning communication and decisions - Married young Couples as peer leaders/changemakers around the issues of family planning for other couples in the society - Behaviour change of the in-laws/ other influential community members around family planning issues in the family/community | 1 |
| Empowering women to plan their pregnancy, childbirth etc is key and more important than accessing one particular service, second the need to explore and improve how to encourage more male participation in pregnancy and childcare - typically our services also today are designed to leave out the man which is unfortunate | 1 |
| Respectful Care during childbirth; empowerment of mothers through individual/ collective information/communication; building effective mothers' collectives | 1 |
| Pregnant mothers satisfaction scores, respectful maternity care.. | 1 |
| Preventing school drop-outs in adolescent girls, delaying age of marriage and improving BMI in girls. Also preventing obesity in adolescent girls and women. Prevention of non-communicable diseases such as diabetes | 1 |
| **Postnatal Care** | 3 |
| Home-based Postnatal care for mothers and newborn for the first 8-10 days Integrated management of maternal health conditions like Anemia, PIDs, STDs etc | 1 |
| Improving post-natal care of mothers- nutrition supplementation, family planning services | 1 |
| Human Resource in Public & Private Sector for Maternal & Newborn Care Midwifery-led Obstetric & Newborn Care Quality of care- antenatal, intrapartum, postnatal | 1 |
| **Health Systems** | 7 |
| health system issues related to maternal health | 1 |
| Health system strengthening for improving maternal outcomes of pregnancy | 1 |
| Involvement of Private Sector for Improving the strategies | 1 |
| NGOs engagement for better penetration in society | 1 |
| Research focusing on maternity benefits for working women (specially informal sector) | 1 |
| service utilization by socio-economic status | 1 |
| **Novel research** | 1 |
| To find out testing of low cost equipment to control PPH | 1 |

Neonatal Health

| **Likert "Other"** | **Ranking "Other"** |
| --- | --- |
| **Low-Birth Weight (n = 2)** | **Care for High Risk Newborns (n = 4)** |
| Community awareness on Neonatal Care specially on LBW (n = 1) | Follow-up care for high-risk newborns (n = 1) |
| Bring down the rate of LBWs (n = 1) | Identification of failure to thrive (n = 1) |
|  | Identification of high-risk pregnancy and follow-up (n = 1) |
|  | Prevention of LBWs (n = 1) |
|  | **Antenatal Care (n = 2)** |
|  | Training of care givers at home in newborn and infant care including exclusive breast feeding (n = 1) |
|  | Awareness of do's and dont's among the mothers (n = 1) |

| **Likert "Other"** | **16** | **Ranking "Other" (n = 12)** | **12** |
| --- | --- | --- | --- |
| Training | 1 | Follow-up care for high-risk newborns | 1 |
| Follow-up care for high-risk newborns | 1 | Training of care givers at home in newborn and infant care including exclusive breast feeding | 1 |
| Colostrum feeding at birth or soon after | 1 | Awareness of do's and dont's among the mothers | 1 |
| Community awareness on Neonatal Care specially on LBW | 1 | Provision of pre-conception care | 1 |
| Diarrhoea & ARI prevention | 1 | nutrition research through newer frameworks such as one health cross sectoral convergence | 1 |
| Provision of pre-conception care | 1 | building capacity of mothers to care for their newborns and infants | 1 |
| nutrition research through newer frameworks such as one health cross sectoral convergence | 1 | Identification of failure to thrive | 1 |
| Identification of failure to thrive | 1 | Assessment of newborn and management based on th individual need | 1 |
| Assessment of every newborn & plan management based on the needs | 1 | Improving preconception and antenatal care for neonatal outcomes | 1 |
| Improving maternal health services for neonatal outcomes | 1 | Identification of high-risk pregnancy and follow-up | 1 |
| Home delivery | 1 | Behavioral and cultural aspects | 1 |
| Strengthening the Service Delivery Points | 1 | Prevention of LBWs | 1 |
| Identification of high-risk pregnancy, and peripartum monitoring. | 1 |  |  |
| Behavioral and cultural aspects | 1 |  |  |
| Early childhood development | 1 |  |  |
| Bring down the rate of LBWs | 1 |  |  |

| **Policies** | **3** |
| --- | --- |
| Influencing the Policy makers to institutionalize ' prioritization' of families for ' enabling to access special care of neonates'(on the criteria of poverty/ marginalized status/ Socio cultural barriers etc. | 1 |
| Enforcing the law to prevent promotion of infant formula. | 1 |
| Public sector /Asha/ AWW linkage in providing community neonatal services | 1 |
| **Postnatal Care** | **7** |
| Care at home of high-risk newborn babies after discharge from the hospital | 1 |
| Community awareness drive for neonatal care specially about LBW children engaging NGOs | 1 |
| Continuation of exclusive breastfeeding and professional/ accessible advice on it | 1 |
| Do we really need oxytocin after delivery? Affect of oxytocin on the baby? | 1 |
| High risk newborn follow-up, Early intervention in newborns | 1 |
| 1. Referral Care for small & sick newborns, with quality 2. Community-Facility linkage for newborn care, including follow-up of high-risk newborns, early screening, detection & intervention | 1 |
| perinatal mortality and still births | 1 |
| **Antenatal Care** | **3** |
| 100% coverage of ANC and 100% institutional delivery | **1** |
| building capacity of mothers to care for their newborns through early classes for them, maternal support groups. today services are geared to ensure health workers care for newborns - instead focus should be on mothers | 1 |
| Pre-natal diagnosis and management | 1 |
| **Health Systems** | **8** |
| health system issues related to child health | 1 |
| Infections | 1 |
| Quality of neonatal care facility | 1 |
| Research into transport to health facilities | 1 |
| ROLE OF SNCUs IN MANAGING NEOTATES | 1 |
| Screening for congenital heart disease, retinopathy of prematurity & metabolic disorders in neonates. | 1 |
| Strengthening the Service Delivery Points | 1 |
| Training and re-training of health workers at all levels, and accountability. | 1 |
| **Family Planning** | **3** |
| Setting right the Mindset / family preference of Gender selection of the preborn. | 1 |
| Engagement of father and in-laws in early home based newborn care | 1 |
| Father and family support and involvement in Newborn Care | 1 |
| **Novel areas** | 3 |
| Reasons for occurrence of Preterm births | 1 |
| Recognition of early signs of development delay  Prevention and management of failure to thrive | 1 |
| Role of age-old customs and practices in newborn care and it's benefits and adverse effects. | 1 |

Infectious Diseases

| **Likert "Other"** | **Ranking "Other"** |
| --- | --- |
| **Respiratory Infections (n = 6)** | **Respiratory Infections (n = 8)** |
| ARI (n = 1) | Respiratory infections- especially pneumonia (n = 1) |
| URI/LRIs (n = 1) | ARI (n = 1) |
| ARI & Pneumonia management. IMNCI (n = 1) | URI/LRI (n = 1) |
| Pneumonia screening and treatment in children (n = 1) | IMNCI / ARI & Pneumonia management (n = 1) |
| Acute Respiratory Illness prevention and management (n = 1) | ARI (n = 1) |
| Acute respiratory infections and pneumonia (n = 1) | Acute Respiratory Illness prevention and management (n = 1) |
|  | Acute respiratory infections and pneumonia (n = 1) |
| **Systems strengthening (3)** | Management of ARI in children (n = 1) |
| capacity building of informal providers ayush doctors | **Hepatitis (n = 4)** |
| Management of childhood illnesses in rural and tribal areas | hepatitis (n = 1) |
| Improving ICDS coverage and utilization. Reasons for stunting and preventing and treatment of stunting at home or in the community. | Hepatitis B (n = 1) |
| **Novel areas (3)** | Hepatitis C (n = 1) |
| We need to work in Selected focused areas | maternal and neonatal screening for hepatitis B (n = 1) |
| Zika |  |
| Data for decision making |  |

| **Likert "Other"** | **17** | **Ranking "Other"** | **32** |
| --- | --- | --- | --- |
| Treatment of anaemia | 1 | Respiratory infections- especially pneumonia | 1 |
| ARI | 1 | Nutritional research | 1 |
| Improving ICDS coverage and utilization. Reasons for stunting and preventing and treatment of stunting at home or in the community. | 1 | ARI | 1 |
| Hygiene awareness | 1 | Chicken pox and small-pox prevention and treatment | 1 |
| Pediatric leprosy | 1 | Immunity booster mission for maternal and neonatal | 1 |
| Hepatitis C | 1 | Hygiene awareness | 1 |
| Capacity building of informal providers ayush doctors | 1 | the change in trend of diseases over time | 1 |
| URI/LRIs | 1 | hepatitis | 1 |
| Zika | 1 | Awareness on disease transmission | 1 |
| Data for decision making | 1 | Hepatitis B | 1 |
| ARI & Pneumonia management. IMNCI | 1 | Hepatitis C | 1 |
| Management of childhood illnesses in rural and tribal areas | 1 | capacity building of informal providers ayush doctors | 1 |
| Pneumonia screening and treatment in children | 1 | skin infections | 1 |
| We need to work in Selected focused areas | 1 | URI/LRI | 1 |
| Acute Respiratory Illness prevention and management | 1 | Any other | 1 |
| Acute respiratory infections and pneumonia | 1 | Zika | 1 |
| Minimizing the exposure of pregnant and prepregnant mothers and young children to 'Risk factors' | 1 | combination of two or more infectious disease | 1 |
|  |  | performance indicators in MCH | 1 |
|  |  | maternal and neonatal screening for hepatitis B | 1 |
|  |  | IMNCI / ARI & Pneumonia management | 1 |
|  |  | ARI | 1 |
|  |  | STI/RTI | 1 |
|  |  | pneumonia screening and treatment | 1 |
|  |  | Infrastructure preparedness | 1 |
|  |  | Management of ARI in children | 1 |
|  |  | We need to work in Selected focused areas | 1 |
|  |  | Role of WASH in prevention of infectious diseases | 1 |
|  |  | Inter departmental convergence for institutionalization | 1 |
|  |  | Acute Respiratory Illness prevention and management | 1 |
|  |  | Acute respiratory infections and pneumonia | 1 |
|  |  | Early childhood development | 1 |
|  |  | minimizing the exposure of Pre pregnnt and pregnant women to risk factors. | 1 |

| **Systems strengthening** | **4** |
| --- | --- |
| 2.Educate and enable communities to adopt' Inf-diseases Appropriate Behaviours as a way of life, at all times. | 1 |
| Preparedness for emerging infections | 1 |
| Preventive Education of Infectious Diseases within community including ASHA, AWW, ANM. | 1 |
| Role of environmental factors such as sanitation, hygiene etc. in infectious | 1 |
| **Health Systems** | **5** |
| Effective management on infectious diseases among children | 1 |
| Health system strengthening - Issues of HMIS, Behaviour change communication, Community empowerment and ownership of programmes | 1 |
| these vary region by region - the focus should be on nutrition exaggerating the impact of these - so ideally it should be integrated management. nutrition when diarrhea and after and so on | 1 |
| We need to work in Selected focused areas | 1 |
| **Infectious Conditions** | **10** |
| Bacterial pneumonia | 1 |
| Diseases like Measles, Rubella and Japanese encephalitis | 1 |
| Emerging infections, antibiotic resistance prevention | 1 |
| Japanese Encephalitis management | 1 |
| MCH outcome and control of malaria and tuberculosis | 1 |
| Measles & Rubella | 1 |
| Neonatal tetanus surveillance | 1 |
| Respiratory infections- especially pneumonia | 1 |
| STI/ RTI | 1 |
| Syphilis | 1 |
| Leprosy | 1 |
| **Novel research** | 3 |
| 1 Predictive research to identify possible New infectious / Resurgence of controlled/ eliminated diseases in coming 3 to 5 yrs. | 1 |
| Bed side identification of organisms and resistance | 1 |
| What is the infection rate in labour room of the country? | 1 |
| **Education/awareness** | 3 |
| awareness about cough etiquettes, hand hygiene, and overall sanitation | 1 |
| Mass community awareness on Hygiene and engagement of NGOs and community organization for such. | 1 |
| The political ruling class needs to be schooled on the importance of public health as a basic prerequisite for nation building. | 1 |
|  |  |
|  |  |

1. **Demographics**

| **Profession** | | | **N = 84 (n, %)** |  | |
| --- | --- | --- | --- | --- | --- |
| Healthcare Worker | | | 23 | (27.38095) | |
| Doctor | | | 20 | (23.80952) | |
| Nutritionist | | | 1 | (1.190476) | |
| Public Health Specialist | | | 1 | (1.190476) | |
| State Programme officer, RCH | | | 1 | (1.190476) | |
| Policy- maker | | | 5 | (5.952381) | |
| Public health research | | | 24 | (28.57143) | |
| Teaching | | | 14 | (16.66667) | |
| Lab research | | | 2 | (2.380952) | |
| Other, specify: | | | 19 | (22.61905) | |
| Academia | | | 3 | (3.571429) | |
| Consultant | | | 3 | (3.571429) | |
| Development | | | 1 | (1.190476) | |
| Donor | | | 2 | (2.380952) | |
| Financial | | | 1 | (1.190476) | |
| Public Health Professional | | | 5 | (5.952381) | |
| Technical Support | | | 1 | (1.190476) | |
|  | | |  |  | |
| **Type of Organization (Primary)** | | | **N = 83 (n, %)** |  | |
| Government | | | 27 | (32.53012) | |
| Non-government | | | 20 | (24.09639) | |
| Private organization | | | 8 | (9.638554) | |
| University | | | 11 | (13.25301) | |
| Other, specify: | | | 17 | (20.48193) | |
| Academics | | | 2 | (2.409639) | |
| Autonomous Premier Institute | | | 1 | (1.204819) | |
| Development agency | | | 1 | (1.204819) | |
| Foundation | | | 1 | (1.204819) | |
| Government Entities | | | 2 | (2.409639) | |
| Private organization | | | 2 | (2.409639) | |
| Research organization | | | 1 | (1.204819) | |
| United Nations | | | 3 | (3.614458) | |
| UNICEF | | | 1 | (1.204819) | |
| WHO | | | 2 | (2.409639) | |
| Missing | | | 1 | (1.204819) | |
| **Primary Area of Work** | | | **N = 84 (n, %)** |  | |
| Biostats/Epidemiology | | | 12 | (14.28571) | |
| Child Health | | | 17 | (20.2381) | |
| Family Planning | | | 1 | (1.190476) | |
| Health Policy | | | 6 | (7.142857) | |
| Infectious Disease | | | 14 | (16.66667) | |
| Maternal Health | | | 7 | (8.333333) | |
| Other, Specify: | | | 27 | (32.14286) | |
| Adolescent Health | | | 1 | (1.190476) | |
| Clinical Research | | | 2 | (2.380952) | |
| Community Health (child focussed) | | | 1 | (1.190476) | |
| Education | | | 1 | (1.190476) | |
| Environmental Health | | | 1 | (1.190476) | |
| Evidence Synthesis | | | 1 | (1.190476) | |
| Geriatrics | | | 1 | (1.190476) | |
| Health Economics | | | 3 | (3.571429) | |
| Health Systems | | | 4 | (4.761905) | |
| Interventions | | | 1 | (1.190476) | |
| Menstrual health (maternal) | | | 1 | (1.190476) | |
| Mental health | | | 1 | (1.190476) | |
| Non-communicable Diseases | | | 1 | (1.190476) | |
| Nutrition | | | 3 | (3.571429) | |
| Occupational Health | | | 1 | (1.190476) | |
| Public Health Planning | | | 2 | (2.380952) | |
| RMNCH+A (maternal health) | | | 1 | (1.190476) | |
| Rural/Tribal Health Outreach | | | 2 | (2.380952) | |
|  | | |  |  | |
| **Number of Years Worked in Primary Area** | | | **N = 82 (n, %)** |  | |
| 1-5 | | | 11 | (13.41463) | |
| 5-10 | | | 16 | (19.5122) | |
| 10-15 | | | 12 | (14.63415) | |
| 15-20 | | | 15 | (18.29268) | |
| 20-25 | | | 13 | (15.85366) | |
| 25-30 | | | 5 | (6.097561) | |
| 30-25 | | | 1 | (1.219512) | |
| 30-35 | | | 2 | (2.439024) | |
| 35-40 | | | 3 | (3.658537) | |
| 40+ | | | 4 | (4.878049) | |
| Missing | | | 2 | (2.439024) | |
|  | | |  |  | |
|  | **N = 84** |  | | |  |
| **City** | **n** | **(%)** | | |  |
| Agartala | 1 | (1.19047619) | | |  |
| Ahmedabad | 1 | (1.19047619) | | |  |
| Alappuzha | 1 | (1.19047619) | | |  |
| Bangalore | 3 | (3.571428571) | | |  |
| Bankura | 2 | (2.380952381) | | |  |
| Bengaluru | 3 | (3.571428571) | | |  |
| Bhubaneswar | 1 | (1.19047619) | | |  |
| Chamarajanagar | 1 | (1.19047619) | | |  |
| Chennai | 1 | (1.19047619) | | |  |
| Delhi | 7 | (8.333333333) | | |  |
| Guwahati | 2 | (2.380952381) | | |  |
| Howrah | 1 | (1.19047619) | | |  |
| Hyderabad | 4 | (4.761904762) | | |  |
| Jodhpur | 1 | (1.19047619) | | |  |
| Kalyani | 1 | (1.19047619) | | |  |
| Kharagpur | 1 | (1.19047619) | | |  |
| Kolkata | 23 | (27.38095238) | | |  |
| Lucknow | 1 | (1.19047619) | | |  |
| Mumbai | 7 | (8.333333333) | | |  |
| New Delhi | 7 | (8.333333333) | | |  |
| Noida | 2 | (2.380952381) | | |  |
| Parganas | 1 | (1.19047619) | | |  |
| Puducherry | 1 | (1.19047619) | | |  |
| Pune | 4 | (4.761904762) | | |  |
| Shimla | 1 | (1.19047619) | | |  |
| Wardha | 1 | (1.19047619) | | |  |

|  | **N = 84** |  |
| --- | --- | --- |
| **State** | **n** | **%** |
| Kerala | 1 | (1.19047619) |
| Assam | 2 | (2.38095238) |
| Delhi | 14 | (16.6666667) |
| Gujarat | 1 | (1.19047619) |
| Himachal Pradesh | 1 | (1.19047619) |
| Jammu and Kashmir | 1 | (1.19047619) |
| Karnataka | 9 | (10.7142857) |
| Maharashtra | 12 | (14.2857143) |
| Odisha | 1 | (1.19047619) |
| Rajasthan | 1 | (1.19047619) |
| Tamil Nadu | 2 | (2.38095238) |
| Telangana | 4 | (4.76190476) |
| Tripura | 1 | (1.19047619) |
| Uttar Pradesh | 3 | (3.57142857) |
| West Bengal | 30 | (35.7142857) |
