# Supplementary material for: Shifting research priorities in maternal and child health in the COVID-19 pandemic era in India: A renewed focus on systems strengthening
Source: PLoS One. 2021 Aug 12;16(8):e0256099. doi: 10.1371/journal.pone.0256099 (PMC8360530; doi:10.1371/journal.pone.0256099)
Supplement: Attachment — Submitted filename: Responses to reviewers comments.docx [file pone.0256099.s003.docx]

Dear Dr. Shet,

Thank you for submitting your manuscript to PLOS ONE. After careful consideration, we feel that it has merit but does not fully meet PLOS ONE’s publication criteria as it currently stands. Therefore, we invite you to submit a revised version of the manuscript that addresses the points raised during the review process.

Considering the reviewers reports and my own reading of the paper, I reached to a decision of minor revision. Looking forward to read the revised version of this paper. 


Please submit your revised manuscript by Jun 26 2021 11:59PM. If you will need more time than this to complete your revisions, please reply to this message or contact the journal office at [plosone@plos.org](mailto:plosone@plos.org). When you're ready to submit your revision, log on to <https://www.editorialmanager.com/pone/> and select the 'Submissions Needing Revision' folder to locate your manuscript file.

Please include the following items when submitting your revised manuscript:

- A rebuttal letter that responds to each point raised by the academic editor and reviewer(s). You should upload this letter as a separate file labeled 'Response to Reviewers'.
- A marked-up copy of your manuscript that highlights changes made to the original version. You should upload this as a separate file labeled 'Revised Manuscript with Track Changes'.
- An unmarked version of your revised paper without tracked changes. You should upload this as a separate file labeled 'Manuscript'.

If you would like to make changes to your financial disclosure, please include your updated statement in your cover letter. Guidelines for resubmitting your figure files are available below the reviewer comments at the end of this letter.

If applicable, we recommend that you deposit your laboratory protocols in protocols.io to enhance the reproducibility of your results. Protocols.io assigns your protocol its own identifier (DOI) so that it can be cited independently in the future. For instructions see: [http://journals.plos.org/plosone/s/submission-guidelines#loc-laboratory-protocols](https://nam02.safelinks.protection.outlook.com/?url=http%3A%2F%2Fjournals.plos.org%2Fplosone%2Fs%2Fsubmission-guidelines%23loc-laboratory-protocols&data=04%7C01%7Ckmehta6%40jhu.edu%7Ca746e5e5bb2f454ef4ec08d9153e58ce%7C9fa4f438b1e6473b803f86f8aedf0dec%7C0%7C0%7C637564179784556691%7CUnknown%7CTWFpbGZsb3d8eyJWIjoiMC4wLjAwMDAiLCJQIjoiV2luMzIiLCJBTiI6Ik1haWwiLCJXVCI6Mn0%3D%7C1000&sdata=5OaW1e6fGUxjGf99o5VfJ5WbNbyCxO75KJPgIlg4yrs%3D&reserved=0). Additionally, PLOS ONE offers an option for publishing peer-reviewed Lab Protocol articles, which describe protocols hosted on protocols.io. Read more information on sharing protocols at [https://plos.org/protocols?utm_medium=editorial-email&utm_source=authorletters&utm_campaign=protocols](https://nam02.safelinks.protection.outlook.com/?url=https%3A%2F%2Fplos.org%2Fprotocols%3Futm_medium%3Deditorial-email%26utm_source%3Dauthorletters%26utm_campaign%3Dprotocols&data=04%7C01%7Ckmehta6%40jhu.edu%7Ca746e5e5bb2f454ef4ec08d9153e58ce%7C9fa4f438b1e6473b803f86f8aedf0dec%7C0%7C0%7C637564179784566691%7CUnknown%7CTWFpbGZsb3d8eyJWIjoiMC4wLjAwMDAiLCJQIjoiV2luMzIiLCJBTiI6Ik1haWwiLCJXVCI6Mn0%3D%7C1000&sdata=esTle15AYwfZND8IkZjToF5TR93fCRLOVI%2B%2FWpVg66s%3D&reserved=0).

We look forward to receiving your revised manuscript.

Kind regards,

Srinivas Goli, Ph.D.
Academic Editor
PLOS ONE

Journal Requirements:

When submitting your revision, we need you to address these additional requirements.

**1. Please ensure that your manuscript meets PLOS ONE's style requirements, including those for file naming. The PLOS ONE style templates can be found at**

<https://journals.plos.org/plosone/s/file?id=wjVg/PLOSOne_formatting_sample_main_body.pdf> and

[https://journals.plos.org/plosone/s/file?id=ba62/PLOSOne_formatting_sample_title_authors_affiliations.pdf](https://nam02.safelinks.protection.outlook.com/?url=https%3A%2F%2Fjournals.plos.org%2Fplosone%2Fs%2Ffile%3Fid%3Dba62%2FPLOSOne_formatting_sample_title_authors_affiliations.pdf&data=04%7C01%7Ckmehta6%40jhu.edu%7Ca746e5e5bb2f454ef4ec08d9153e58ce%7C9fa4f438b1e6473b803f86f8aedf0dec%7C0%7C0%7C637564179784566691%7CUnknown%7CTWFpbGZsb3d8eyJWIjoiMC4wLjAwMDAiLCJQIjoiV2luMzIiLCJBTiI6Ik1haWwiLCJXVCI6Mn0%3D%7C1000&sdata=rSQNdSKZZwiTaYPFLipExSbD55WXwFLcsNcTC5Wd2Qk%3D&reserved=0)

 We have ensured that the revised manuscript meets PLOS One’s style requirements.

**2. Please review your reference list to ensure that it is complete and correct. If you have cited papers that have been retracted, please include the rationale for doing so in the manuscript text, or remove these references and replace them with relevant current references. Any changes to the reference list should be mentioned in the rebuttal letter that accompanies your revised manuscript. If you need to cite a retracted article, indicate the article’s retracted status in the References list and also include a citation and full reference for the retraction notice.**

 We have reviewed the reference list in the revised manuscript to ensure that it is complete, current and meets PLOS One’s requirements.

**3. Please include additional information regarding the survey or questionnaire used in the study and ensure that you have provided sufficient details that others could replicate the analyses. For instance, if you developed a questionnaire as part of this study and it is not under a copyright more restrictive than CC-BY, please include a copy, in both the original language and English, as Supporting Information.**

We have attached the questionnaire used for this survey during the resubmission process.

4. **During our internal checks, the in-house editorial staff noted that you conducted research or obtained samples in another country. Please check the relevant national regulations and laws applying to foreign researchers and state whether you obtained the required permits and approvals. Please address this in your ethics statement in both the manuscript and submission information. In addition, please ensure that you have suitably acknowledged the contributions of any local collaborators involved in this work in your authorship list and/or Acknowledgements. Authorship criteria is based on the International Committee of Medical Journal Editors (ICMJE) Uniform Requirements for Manuscripts Submitted to Biomedical Journals - for further information please see here:** <https://journals.plos.org/plosone/s/authorship>

This study was reviewed by the Johns Hopkins Bloomberg School of Public Health (JHSPH) Institutional Review Board (IRB) which classified the proposed activities as “key informants research”, and therefore determined to be exempt from human subjects research oversight. As a result, as per relevant national regulations, we did not need to obtain any additional approvals to administer this survey to stakeholders engaged in maternal and child health in India. This information has been added to the ethics statements as requested. We further acknowledge our local collaborators, from the Indian Academy of Preventive and Social Medicine (IAPSM) in the acknowledgements section of our revised manuscript.

5. **Thank you for stating the following in the Acknowledgments Section of your manuscript:**

**'The authors acknowledge funding provided by the Johns Hopkins Maternal and Child Health
Center - India, and support from the Indian Association of Preventive and Social Medicine
(IAPSM).'**

**We note that you have provided funding information that is not currently declared in your Funding Statement. However, funding information should not appear in the Acknowledgments section or other areas of your manuscript. We will only publish funding information present in the Funding Statement section of the online submission form.**

**a. Please remove any funding-related text from the manuscript and let us know how you would like to update your Funding Statement. Currently, your Funding Statement reads as follows:**

**'The funders had no role in study design, data collection and analysis, decision to publish, or preparation of the manuscript.'**

We have removed references to funding information in the acknowledgements section, and would like to revise the funding statement thus:

“The authors acknowledge funding provided by the Johns Hopkins Maternal and Child Health
Center - India, and support from the Indian Association of Preventive and Social Medicine
(IAPSM). The funders had no role in study design, data collection and analysis, decision to publish, or preparation of the manuscript”.

**b. Please include your amended statements within your cover letter; we will change the online submission form on your behalf.**

We have included the above amended statement within our cover letter submitted at the time of submission of the revised manuscript.

**Additional Editor Comments:

Considering the reviewers reports and my own reading of the paper, I reached to a decision of minor revision. Looking forward to read the revised version of this paper.**


[Note: HTML markup is below. Please do not edit.]

Reviewers' comments:

Reviewer's Responses to Questions

**Comments to the Author**

1. Is the manuscript technically sound, and do the data support the conclusions?

The manuscript must describe a technically sound piece of scientific research with data that supports the conclusions. Experiments must have been conducted rigorously, with appropriate controls, replication, and sample sizes. The conclusions must be drawn appropriately based on the data presented.

Reviewer #1: Partly

Reviewer #2: Yes

Reviewer #3: Yes

2. Has the statistical analysis been performed appropriately and rigorously?

Reviewer #1: N/A

Reviewer #2: Yes

Reviewer #3: Yes

3. Have the authors made all data underlying the findings in their manuscript fully available?

The [PLOS Data policy](https://nam02.safelinks.protection.outlook.com/?url=http%3A%2F%2Fwww.plosone.org%2Fstatic%2Fpolicies.action%23sharing&data=04%7C01%7Ckmehta6%40jhu.edu%7Ca746e5e5bb2f454ef4ec08d9153e58ce%7C9fa4f438b1e6473b803f86f8aedf0dec%7C0%7C0%7C637564179784576687%7CUnknown%7CTWFpbGZsb3d8eyJWIjoiMC4wLjAwMDAiLCJQIjoiV2luMzIiLCJBTiI6Ik1haWwiLCJXVCI6Mn0%3D%7C1000&sdata=LKBHDYG%2BaAjuBxyOjWYDS%2BHH%2FTC4nA32KoN85agAhzI%3D&reserved=0) requires authors to make all data underlying the findings described in their manuscript fully available without restriction, with rare exception (please refer to the Data Availability Statement in the manuscript PDF file). The data should be provided as part of the manuscript or its supporting information, or deposited to a public repository. For example, in addition to summary statistics, the data points behind means, medians and variance measures should be available. If there are restrictions on publicly sharing data—e.g. participant privacy or use of data from a third party—those must be specified.

Reviewer #1: No

Reviewer #2: No

Reviewer #3: No

4. Is the manuscript presented in an intelligible fashion and written in standard English?

PLOS ONE does not copyedit accepted manuscripts, so the language in submitted articles must be clear, correct, and unambiguous. Any typographical or grammatical errors should be corrected at revision, so please note any specific errors here.

Reviewer #1: No

Reviewer #2: Yes

Reviewer #3: Yes

5. Review Comments to the Author

Please use the space provided to explain your answers to the questions above. You may also include additional comments for the author, including concerns about dual publication, research ethics, or publication ethics. (Please upload your review as an attachment if it exceeds 20,000 characters)

**Reviewer #1: How shifting research priorities in MCH could be the new focus on systems strengthening? It would be beneficial to explain or include in the introduction section as this could direct your focus. Perhaps this could highlight or give a better picture of overall intention of doing this survey?**

Thank you for this suggestion. We have added these points in the introduction section of the revised manuscript, Briefly, these lines capture the essence of the findings of similar previous studies (conducted prior to the COVID-19 pandemic). Further, we clarify that there was a knowledge gap regarding the priority areas in maternal and child health research in India during the pandemic era, necessitating this rapid assessment, which then revealed the new focus of the shifting research priorities in MCH to be predominantly health systems strengthening needs.

**Any form of survey technique was used to get consensus from the stakeholders or key participants?**

Our survey was jointly developed by researchers at the Johns Hopkins Maternal and Child Health Center in India and key stakeholders at Indian institutions engaged in maternal and child health that included the Public Health Foundation of India, All India Institute of Medical Sciences, Kalyani, Postgraduate Institute of Medical Education and Research, Indian Institute of Technology, Kharagpur and Piramal Swasthya Management and Research Institute. Research domains were identified through a review of previous survey results, and literature on global priorities in maternal newborn and child health in consultation with key stakeholders in important Indian institutions. All key stakeholders reviewed the survey and research domains included, and provided input that was incorporated; the final survey was derived by consensus. As this was intended as a rapid assessment, we did not conduct iterative consensus building exercises, and this has been clarified in the Methods section of the revised manuscript.

**Do the survey questions developed be tested or validated? And how do the stakeholders were approached asking them to take part in a survey. Any form of sampling technique was used?**

Since we aimed to conduct a rapid assessment to identify top research priorities in maternal and child health, we piloted the survey questions among key stakeholders prior to disseminating the survey among potential respondents. The survey tool was novel and was not validated. Key stakeholders working in the field of MCH in India were identified through desk reviews, web-based searches, dissemination in professional networks such as the Indian Association of Preventive and Social Medicine, Indian Public Health Association, Indian Academy of Pediatrics and the Federation of Obstetric and Gynecological Societies of India, and through collaborators. It was a convenience sample based on a snowball sampling strategy. We have added this information to the methods section in the revised manuscript. We acknowledge in the limitation section the potential presence of bias, but have taken care to include a large and diverse respondent population.

**Authors have mentioned how they analyzed the survey response. But who and how**

We have discussed the methodology we employed for data analysis in greater detail in the revised manuscript. Specifically, we have included the formula we used to calculate the “average ranking score” and substantiate this further with an example to make it easy for our readers. Ranking analysis was conducted by three authors, and we have added these details to the methods section of the revised manuscript.

**Participant characteristics: why 84 respondents from 15 states in India. Should it be the main stakeholders? or does it refers to stakeholders from each state in India. Has it been mentioned?**

All our respondents were key stakeholders working in the space of maternal and child health in India. They were located in 15 states and 2 union territories in India. Thank you for this suggestion, we have added this information to the participant characteristics in the results section of the revised manuscript.

**I would say the primary area of work for others, 16 is many. Would it be any way to regrouped the primary area of work, so it provides more representative towards those work related to MCH?**

We took a closer look at the primary area of work listed by the respective respondent, and have updated table 1 in the revised manuscript. Following appropriate regrouping, as well as the addition of two new categories, namely, environmental and occupational health, and clinical research, we are now left with only 4 areas listed under “other”. This will make for clearer understanding for the readers.

**Overall ranking of research priorities: the word 'concurrent' to explain the overall ranking exercise may not be suitable, suggest to rewrite**

We have replaced the word “concurrent’ with “in agreement”, signifying that the priority research domains identified by the Likert scale importance assignment and overall ranking exercises were in agreement in all domains, except the infectious disease domain.

**Authors have listed out the common themes identified as other research priorities. However, these rather similar to priorities identified in the 6 domains except those considered as not important as system strengthening priorities.**

We agree that the “other research priorities” identified by the respondents are similar to the broader sub-domain themes, and fall within the wider area of systems strengthening. We clarify this further in the results section of the revised manuscript, and have further added other non-systems strengthening themes identified by the respondents in the revised manuscript.

**The second last paragraph of discussion: this paragraph mainly described or explained how countries responding on covid-19 which found not appropriate. How this relate to the top research priorities? It may be valuable to include the steps further, would the results of this survey be disseminated? to enhance systems strengthening require a coorporation between researchers and stakeholders. So, would it be any engagement with the stakeholder afterwards?**

These are very helpful observations. In our study, we found that across various domains of maternal and child health, the overall focus was on strengthening existing services and service delivery mechanisms during the COVID-19 pandemic era. As a real-world example, in the penultimate paragraph we discuss how certain countries that invested heavily on their health systems early in the course of the pandemic, experienced early success in coronavirus control, substantiating the fact that health system strengthening is of utmost importance, as was identified by the respondents in our study.

**How the general recommendations (Figure 2) arised?**

The general recommendations in figure 2 have been derived from the World Health Organization’s report on research for universal health coverage, and they have also been informed by the findings of our study and discussions with key stakeholders. We have added these details to the discussion section of our revised manuscript, and also to the figure’s legend.

**Reviewer #2: Thank you for sharing the paper for review. The topic is important and relevant.

General comments:**

**The authors emphasized health system strengthening in relation to COVID-19 pandemic, which only highlights the shortcomings of the existing health system facing a crisis. There was neither elaboration on why the earlier studies on research priority setting paid limited or no attention to health system domains for priority setting nor there were any indications that these priorities will be institutionalized irrespective of the COVID-19 or other emerging diseases or crisis. Some statements like importance of public health systems or importance of public-private partnership are made with limited supportive evidence. The reader would benefit from more evidence supporting statements. In addition, it would be informative for reader to provide rationale for six domains for research priority and how they link to health system strengthening, including their operational definition of health system strengthening.

I recommend for publication subject to revision and addressing comments raised.**

In the introduction section of the revised manuscript, we explain how results from similar research priority setting exercises, undertaken both globally and nationally had identified research priorities that were mostly related to implementation research; several of which pertain to health systems strengthening as well. However, these exercises had also identified areas of novel “discovery” research, and data were lacking regarding the relevance of such priorities during the era of an unprecedented pandemic. We undertook this rapid assessment to further understand and inform this data gap, and found that the overall focus for research priorities in MCH in India during the COVID-19 pandemic is on strengthening existing services and service delivery, rather than novel research.

Since we designed our rapid assessment to determine research priorities in maternal and child health in India during the COVID pandemic, it would be difficult for us to predict how the identified priorities would change if the reference for COVID-19 would not be made. While it is likely that the focus will continue to remain on health systems strengthening, given that the pandemic has exposed glaring loopholes in health systems, this question motivates us to conduct a follow up exercise to identify top priorities in maternal and child health research once the pandemic subsides.

With regards to the importance of public health systems and public-private partnerships, we have added relevant references to substantiate the statement.

Following the reviewer’s observations and suggestions, in the revised manuscript, we have further explained how the six research domains were identified, and in the results and discussion sections, we address how the top priorities identified are related to health systems strengthening. We used the WHO’s operational definition for health systems strengthening to thematically analyze responses that would be categorized into the broader theme of “health systems strengthening”; and have included this information in the methods section of the revised manuscript.

**Method:

a) Representative sample – The paper would benefit by describing how various stakeholders were selected to have representative sample, despite being a convenient sample.**

In the methods section of the revised manuscript, we have now discussed our sampling strategy in greater detail. To clarify, stakeholders were identified across various domains of maternal and child health, including those primarily engaged in academia, or specific research areas such as health systems, policy and economics, nutrition, and tribal health. Since this was a rapid assessment, we employed convenience sampling, based on a snowball sampling strategy, and we have added these details in the methods section of the revised manuscript.

**b) Sample size: There is no mention of how sample size was calculated or what was the expected response rate? There is no justification given on total number of respondents (N-84) is good for generalizability of results. The authors could elaborate further on it.**

Since this was a rapid assessment, we did not target a specific sample size; we analyzed responses from this that we received until the closure of the online survey. We have added this information to the methods section of the revised manuscript, and further acknowledge this approach as a limitation in the discussion section of our revised manuscript.

**c) Sampling: No sampling design is mentioned. If it is a convenience sampling, it should still be mentioned, and a justification would be helpful.**

We have described our sampling strategy in greater detail in the revised manuscript. To clarify, stakeholders were identified across various domains of maternal and child health, including those primarily engaged in academia, or specific research areas such as health systems, policy and economics, nutrition, and tribal health. Since this was a rapid assessment, we employed convenience sampling, based on a snowball sampling strategy, and we have added these details in the methods section of the revised manuscript.

**Data analysis:
a)** **The authors could provide some information on how they increased the reliability and validity of the collected data. Were there any significant differences in ranking by respondents’ characteristics? Were there regional variations in responses when 15 states are grouped into regions? These comparative analyses could help in shedding light on reliability and validity of the data as well their generalization.**

The results obtained here were obtained from a rapid assessment to identify top research priorities in maternal and child health across India during the COVID-19 pandemic, and included 84 expert respondents from across 15 states and 2 Union Territories in India. While all the regions of India had some representation, the sample size was small and may preclude assessments of rankings by region. However, this is a valid comment by the reviewer, and we examined our data to see if there were regional variations, and unsurprisingly we did not find any significant differences. We will explore if differences exist in future work. We acknowledge these limitations in the Discussion section of the manuscript, advising caution about generalization of these results as regional variations in ranking of priorities may not have been captured due to the small sample size of survey respondents.

**b) The reader would benefit with a specific example of a domain on how formula of average ranking score is applied (Table 2) rather than just describing the formula.**

We have included an example to illustrate how the average ranking score was calculated in the methods section of the revised manuscript. Specifically, in this method, sub-domain research priorities that were ranked as “1” would receive the largest weight depending on how many sub-domain research priorities were present in each domain. For example, in the vaccine preventable diseases domain, if individuals ranked “Strengthening the public health workforce” as their “1” priority, the weight would be “10” since there were 10 sub-domain research priorities listed underneath this domain. If individuals ranked this sub-domain research priority as “2”, then the accompanying weight would be “9”, and so on. The weights were then multiplied by the count of how many respondents ranked the sub-domain research priority as “1”, “2”, and so on. The total sums of all weighted scores were then divided by the total number of respondents for that sub-domain research priority. Thus, the average ranking score of each sub-domain research priority was then calculated using the formula,

Average ranking score = (x_1_w_1_ + x_2_w_2_ + x_3_w_3_ ... x_n_w_n_)/ Total response count

Where*,* w = weight of ranked position, and x = response count for each rank.

**a) The authors could elaborate the added value of their rapid method over Child Health and Nutrition Research Initiative (CHNRI) method for research priority setting and why it should be used over CHNRI method. They can add other methods for comparison as well.**

In the discussion section of the revised manuscript, we mention other methodologies used to identify top research priorities, and describe why we chose our rapid method over the CHNRI method in the discussion section of the revised manuscript. Other research priority setting methodologies such as the CHNRI method, Combined Approach Matrix method, the Essential National Health Research (ENHR) method, Council on Health Research and Development (COHRED) method, the James Lind Alliance method and the Delphi method, are comprehensive methods, but they involve iterative processes which require substantial time and resources to complete. We therefore adopted our rapid method since we aimed to undertake a brisk assessment to identify key priorities for public health research in MCH in India within the context of the COVID-19 pandemic.

**b) The author could clarify if COVID19 was not the reference point then: a) will be the priorities remain the same or be different as reflected by other studies? b) How valid will be the findings if the reference to COVID 19 is not made especially for health system strengthening research?**

We designed our rapid assessment to determine research priorities in maternal and child health in India during the COVID-19 pandemic, with the goal of understanding what, if any, changes in research priorities were perceived by the larger scientific and public health community. If COVID-19 were not a central event during the period of this research, or if this research were conducted prior to the pandemic, it is likely that our findings would be similar to previous studies to identify research priorities in maternal and child health that were conducted prior to the COVID-19 pandemic by Arora et al, 2017 and Souza et al, 2014, that mainly identified priorities that pertained to implementation sciences and innovation.

It is likely that as the pandemic has exposed gaps and weaknesses in the health systems, there is a heightened awareness that these gaps need to be addressed, and hence we believe our findings from this rapid assessment carry immense relevance and urgency.

**c) Health system resilience was mentioned in relation to a study (Page 22), as “...has exposed glaring loopholes in health systems, and has demonstrated that countries’ response to pandemics is ultimately dependent on the resilience of their health systems (17).” However, the authors did not explain why resilience had not been made part of research domains for research priority ranking. Given the debate whether the resilience is an emergent property of the system arising from the interactions of the systems components or a health system capacity, has important policy, program, and research implications, therefore, a rationale exists for putting it for research priority setting and ranking. The authors could clarify their perspective for not considering resilience as a research priority category.**

We agree with the reviewer that health system resilience would have been a domain area to be considered. At the time of the design of the survey, we considered specific topic areas rather than cross-cutting concepts such as health system resilience. Our cross-sectional survey, conducted a few months into the COVID-19 pandemic identified that focusing on research on strengthening existing health systems and service delivery was most important for maternal and child health during the ongoing pandemic. Since health system resilience is the ability to prepare for, manage (absorb, adapt and transform) and learn from shocks such as pandemics, it is likely that a follow up exercise conducted during the post-pandemic period would be well positioned to identify research priorities pertinent to health system resilience. This comment has prompted us to plan to explore health systems resilience further in our future work.

**d) There is no discussion on how these recommendations based on survey will be disseminated to relevant authorities for policy and program development and further research.**

Our rapid assessment to identify top priorities in maternal and child health research in India during the COVID-19 pandemic era has led to important learnings, and we have shared the results of our study with the stakeholders who participated in the study. In addition, we have shared it with the leadership of the premier body of public health professionals in India, the Indian Academy of Preventive and Social Medicine (IAPSM). We hope that by publishing our findings in a reputed journal such as PLoS One would enhance our ability to disseminate these important findings to relevant authorities for policy and program development and further research.

**e) The rationale for how India’s research priorities are relevant for emerging economies need further elaboration/justification.**

Conducting research priority setting exercises is usually an expensive and time-consuming process, and difficult to conduct in other low- and middle-income (LMIC) settings, especially during the ongoing pandemic. The findings of our rapid assessment to determine top research priorities in maternal and child health in India have identified systems strengthening as most important during the pandemic. Other emerging economies such as Sri Lanka and Bangladesh, that have strong primary health systems have fared much better in the pandemic, especially during the second wave, although these data are still emerging. Given the similarities in health systems, populations served and limited resources across other LMIC settings, we believe that policymakers and stakeholders from other emerging economies could also benefit from these findings.

**Reviewer #3: The paper reports on research priorities for MCH in India following the COVID-19 pandemic. The study used CNHR approach to summaries research priorities and input was sought from research, academia, policy makers, and programmes. The study found research on health system strengthening and delivery of existing intervention received highest ranking. The findings are line with expectations during the pandemic when there we few novel interventions that we thought efficacious at the time of the study and optimization of existing interventions are/were most beneficial. The paper is well written and arrives at appropriate conclusions.**

**Minors Comment
• In the discussion the authors should comment on why research on COVID-19 vaccines did not receive high priority ranking. This is likely because most did not expect an efficacious vaccine to be developed within a year of the pandemic and research could add this to their discussion section.**

We agree with the reviewer and are equally surprised to note that research on COVID-19 vaccines did not receive high priority ranking. The most likely reasons are that expectations were low for having a fully developed vaccine within less than a year of virus discovery. We have discussed this further in the discussion section of our revised manuscript.

**• The authors should acknowledge potential limitations to the research methodology especially on the self-selected nature of responders, which may bias findings.**

We discuss the limitations of our study in the discussion section of the revised manuscript, and specifically allude to selection bias, as has been rightly pointed out.

6. PLOS authors have the option to publish the peer review history of their article ([what does this mean?](https://nam02.safelinks.protection.outlook.com/?url=https%3A%2F%2Fjournals.plos.org%2Fplosone%2Fs%2Feditorial-and-peer-review-process%23loc-peer-review-history&data=04%7C01%7Ckmehta6%40jhu.edu%7Ca746e5e5bb2f454ef4ec08d9153e58ce%7C9fa4f438b1e6473b803f86f8aedf0dec%7C0%7C0%7C637564179784576687%7CUnknown%7CTWFpbGZsb3d8eyJWIjoiMC4wLjAwMDAiLCJQIjoiV2luMzIiLCJBTiI6Ik1haWwiLCJXVCI6Mn0%3D%7C1000&sdata=93bh4jFVwyKySsTehcA4EiZvdV1gk5GaeQpzgWfmAD8%3D&reserved=0)). If published, this will include your full peer review and any attached files.


If you choose “no”, your identity will remain anonymous but your review may still be made public.


**Do you want your identity to be public for this peer review?** For information about this choice, including consent withdrawal, please see our [Privacy Policy](https://nam02.safelinks.protection.outlook.com/?url=https%3A%2F%2Fwww.plos.org%2Fprivacy-policy&data=04%7C01%7Ckmehta6%40jhu.edu%7Ca746e5e5bb2f454ef4ec08d9153e58ce%7C9fa4f438b1e6473b803f86f8aedf0dec%7C0%7C0%7C637564179784586681%7CUnknown%7CTWFpbGZsb3d8eyJWIjoiMC4wLjAwMDAiLCJQIjoiV2luMzIiLCJBTiI6Ik1haWwiLCJXVCI6Mn0%3D%7C1000&sdata=zPjl7NSSlhvXE7KgeHNI1W45EA0CuE107w7682DGImY%3D&reserved=0).

Reviewer #1: No

Reviewer #2: No

Reviewer #3: **Yes:** Samuel Akech

[NOTE: If reviewer comments were submitted as an attachment file, they will be attached to this email and accessible via the submission site. Please log into your account, locate the manuscript record, and check for the action link "View Attachments". If this link does not appear, there are no attachment files.]

While revising your submission, please upload your figure files to the Preflight Analysis and Conversion Engine (PACE) digital diagnostic tool, [https://pacev2.apexcovantage.com/](https://nam02.safelinks.protection.outlook.com/?url=https%3A%2F%2Fpacev2.apexcovantage.com%2F&data=04%7C01%7Ckmehta6%40jhu.edu%7Ca746e5e5bb2f454ef4ec08d9153e58ce%7C9fa4f438b1e6473b803f86f8aedf0dec%7C0%7C0%7C637564179784596675%7CUnknown%7CTWFpbGZsb3d8eyJWIjoiMC4wLjAwMDAiLCJQIjoiV2luMzIiLCJBTiI6Ik1haWwiLCJXVCI6Mn0%3D%7C1000&sdata=t%2Fin6yxj6JtWRT2iPcE6becrXTjnyotQ3ywelTNvzi4%3D&reserved=0). PACE helps ensure that figures meet PLOS requirements. To use PACE, you must first register as a user. Registration is free. Then, login and navigate to the UPLOAD tab, where you will find detailed instructions on how to use the tool. If you encounter any issues or have any questions when using PACE, please email PLOS at [figures@plos.org](mailto:figures@plos.org). Please note that Supporting Information files do not need this step.
